# Sensitized Photocatalytic CO_2_ Reduction With Earth Abundant 3d Metal Complexes Possessing Dipicolyl-Triazacyclononane Derivatives

**DOI:** 10.3389/fchem.2021.751716

**Published:** 2021-09-30

**Authors:** Martin Obermeier, Fabian Beckmann, Raoul S. Schaer, Oliver S. Wenger, Matthias Schwalbe

**Affiliations:** ^1^ Institute of Chemistry, Humboldt-Universität zu Berlin, Berlin, Germany; ^2^ Department of Chemistry, Universität Basel, Basel, Switzerland

**Keywords:** photocatalysis, carbon dioxide reduction, 3d metal complexes, electron transfer, sulfur containing ligand

## Abstract

Complexes based on nitrogen and sulfur containing ligands involving 3d metal centers are known for the electrocatalytic reduction of CO_2._ However, photocatalytical activation has rarely been investigated. We herein present results on the light-driven CO_2_ reduction using either Ir(dFppy)_3_ [**Ir**, dFppy = 2-(4,6-difluorophenyl)pyridine] or [Cu(xant)(bcp)]^+^, (**Cu**, xant = xantphos, bcp = bathocuproine) as photosensitizer in combination with TEA (triethylamine) as sacrificial electron donor. The 3d metal catalysts have either dptacn (dipicolyl-triazacyclononane, **L** ^**N3**^) or dpdatcn (dipicolyl-diazathiocyclononane, **L** ^**N2S**^) as ligand framework and Fe^3+^, Co^3+^ or Ni^2+^ as central metal ion. It turned out that the choice of ligand, metal center and solvent composition influences the selectivity for product formation, which means that the gaseous reduction products can be solely CO or H_2_ or a mixture of both. The ratio between these two products can be controlled by the right choice of reaction conditions. With using **Cu** as photosensitizer, we could introduce an intermolecular system that is based solely on 3d metal compounds being able to reduce CO_2_.

## Introduction

Due to the fact that the global energy demand is projected to increase, there will still be a high necessity of usage of fossil fuels over the next couple of decades, as the development of renewable energy sources cannot adjust with the same speed (Sönnchen, 2020). This mismatch will not only cause problems due to an increase in the amount of greenhouse gases in the atmosphere, which are highly responsible for the climate change (e.g. methane, CO_2_), but also raises the need to search for other alternative energy resources (Aresta, 2010; Ringsmuth et al., 2016). Therefore one of the key answers to face both problems is the conversion of CO_2_ to liquid fuels (Benson et al., 2009; Ma et al., 2009). This process is forecast to have a positive impact on the global greenhouse gas balance by recycling prior emitted CO_2_. Due to the inertness of CO_2_ - both thermodynamically and kinetically - different strategies need to be developed to optimize the conversion to more useful C1 building blocks (Schrag, 2007). One option is using homogeneous catalysts instead of heterogeneous catalysts (Kumar et al., 2016) as often done by industry. One major advantage is the higher variety of spectroscopic techniques that can be used to understand the mechanistic features and, hence, assist in finding the correlation between structure and catalytic activity (Francke et al., 2018). Significant progress has been made for the CO_2_ to CO reduction with catalytic systems based on heavier transition metals such as rhenium or ruthenium (Tamaki et al., 2013; Tamaki et al., 2015; Gotico et al., 2018). However, these metals are expensive and rare, which leads to the need of finding complexes based on earth-abundant metals to perform the activation and conversion of CO_2_ (Takeda et al., 2017; Dalle et al., 2019).

As often in modern chemistry, inspiration can be obtained from nature. 3d metal-based enzymes with complex frameworks are known to activate stable small molecules. One of the best studied enzymes is carbon dioxide dehydrogenase (CODH) – capable of oxidizing CO to CO_2_ and *vice versa.* The catalytically active C-cluster is composed of a bimetallic Ni-Fe center in a nitrogen/sulfur rich environment (Lubitz et al., 2014; Esselborn et al., 2016; Benvenuti et al., 2020; Ghosh et al., 2020). Beside molecular Ni and Fe complexes, systems containing Co were found to show high electro- as well as photo-catalytic activity towards CO_2_ reduction by several groups (Tamaki and Ishitani, 2017; Liu et al., 2018). Light-activated processes have the charm, that the necessary activation energy can be harvested from the sun as a limit less power source, which makes the process economical and chemical ultimate efficient. By adding a photosensitizer (PS), which transforms photonic energy into chemical energy, sunlight can be used directly to provide the necessary energy for the CO_2_ reduction (Thoi et al., 2013; Chan et al., 2015; Guo et al., 2016). Photochemical CO_2_ reduction can thus be considered a crucial part of artificial photosynthesis.

Apart from the nature of the metal, the ligand environment is also of great importance. For example, Ni(II) complexes – when having π-donor atoms such as S – are more likely to show activity towards CO_2_ reduction, due to the less negative Ni^II/I^ redox potential compared to Ni complexes with just N-donor atoms (Chen et al., 2014). Interestingly, studies on ligands with sulfur donor atoms are rare. The Ni dithiacyclam complex **N**_**2**_ **S**_**2**_ **Ni** (N_2_S_2_ = 1,8-Dithia-4,11-diazacyclotetradecane) was the first of its kind to be investigated in the electrocatalytic CO_2_ activation. Although better catalytic properties than the [Ni(cyclam)]^2+^ congener were observed (cyclam = 1,4,8,11-tetraazacyclotetradecane), it was revealed that the complex seems to be instable after prolonged electrolysis time (Gerschel et al., 2019; Iffland et al., 2020).

**SCHEME 1:**
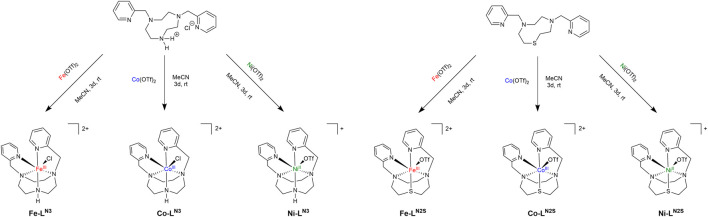
Reaction scheme and picture of the complexes **M-L^N3^** (with M = Fe, Co, Ni; left) and **M-L^N2S^** (with M = Fe, Co, Ni; right) with **L^N3^** being dipicolyl-triazacyclononane and **L^N2S^** being dipicolyl-diazathiacyclononane.

Kojima et al. reported about a heterodinuclear nickel magnesium complex, in which the Ni ion resides in a S_2_N_2_ ligand sphere and two noncoordinating pyridine substituents can bind to a Lewis-acidic metal ion. The heterodinuclear Ni-Mg complex was successfully applied in photocatalytic CO_2_ to CO transformation using [Ru (bpy)_3_]^2+^ (bpy = 2,2′-bipyridine) as photosensitizer (Hong et al., 2017; Hong et al., 2019). Further examples investigating the photocatalytic properties of 3d metal complexes with sulfur donor atoms in the ligand sphere are missing. Hence, we started out to synthesize a series of complexes with earth abundant transition metals (Fe, Co and Ni) and either a macrocyclic sulfur free or sulfur containing ligand (Scheme 1). Herein we present their photocatalytic activity in the presence of different photosensitizers, **Ir** ([Ir(dFppy)_3_], dFppy = 2-(4,6-difluorophenyl)pyridine) and **Cu** ([Cu(xant)(bcp)]PF_6_, xant = xantphos, bcp = bathocuproine).

## Discussion and Results

### Synthesis and Characterization

The complexes were prepared by first synthesizing the corresponding ligands 1,4-di(picolyl)-1,4,7-triazacyclononane **L** ^**N3**^ and 1-thia-4,7-di(picolyl)-diazacyclononane **L** ^**N2S**^. We followed published procedures with slight adjustments (see also the Supporting Information). Briefly, **L** ^**N3**^ was prepared beginning with the tosylation of diethylenetriamine and glycol (Friscourt et al., 2012). The next step was a Cs_2_CO_3_ catalyzed macrocyclization using the tosylated compounds to form tri-tosyl-triazacyclononane (ts_3_-tacn) (Cao et al., 2010). Partial detosylation of the formed macrocycle using HBr in acetic acid resulted in the formation of ts-tacn (Cao et al., 2010), which was transformed into dipicolyl-tosyl-tacn using two equivalents of 2-picolylchloride hydrochloride in a base-catalyzed S_N_2 reaction (Stavila et al., 2008). To obtain the desired ligand **L** ^**N3**^ the remaining tosyl protecting group was cleaved using conc. H_2_SO_4_ (Luk’yanenko et al., 1990; Stavila et al., 2008). **L** ^**N2S**^ was prepared in a similar fashion: First N,N’-bis-tosyl-bis(2-aminoethyl)sulfide was synthesized *via* sodium ethoxide driven S_N_2 reaction of cysteamine hydrochloride and 2-chloroethylamine hydrochloride (Wilson, 2007), followed by tosylation as described earlier for **L** ^**N3**^ (Friscourt et al., 2012). The macrocyclization was started with LiOH (2.5% in water) in toluene and using NaBu_4_Br as phase-transfer catalyst (Wilson, 2007). To obtain **L** ^**N2S**^ a detosylation and substitution with 2-picolylchloride hydrochloride was performed (Chak et al., 1994; Wilson, 2007). Both proligands were used immediately to obtain the complexes, due to sensitivity towards oxidation. Hence it was not possible to store the proligands for longer time periods.

For both compounds, **L** ^**N3**^ and **L** ^**N2S**^, the analytical data nonetheless agreed with those published earlier. Metal complexation could be achieved by reaction of the corresponding ligand with an equimolar amount of simple metal salt, e.g., metal triflates M(OTf)_2_ (M = Fe, Co, Ni). Work-up of the reaction mixture was done under air atmosphere leading to oxidation of the iron and cobalt compounds. Purification of the crude product was obtained *via* washing with CH_2_Cl_2_ to remove excess of ligand and washing with toluene (Fe(OTf)_2_ and Co(OTf)_2_) or THF (Ni(OTf)_2_) to remove unreacted metal salt. The desired complexes were obtained as solids, which were redissolved in a small amount of MeCN and precipitated using Et_2_O. Since **L** ^**N3**^ was synthesized as hydrochloride salt (stemming from column chromatographic purification using dichloromethane), **Fe-L** ^**N3**^ and **Co-L** ^**N3**^ were obtained as complexes with one chloride ligand and two uncoordinated triflate counter anions, which could be verified by mass spectrometry and X-ray diffractometry. **Ni-L** ^**N3**^ was isolated as a chloride free complex by precipitating AgCl using Ag(OTf). The complexes **M-L** ^**N2S**^ were prepared in the same fashion giving yields in the range of 44–50%. All complexation reactions should be done soon after preparation of the proligands, due to sensitivity against oxidation of the compounds **L** ^**N3**^ and **L** ^**N2S**^.

**FIGURE 1:**
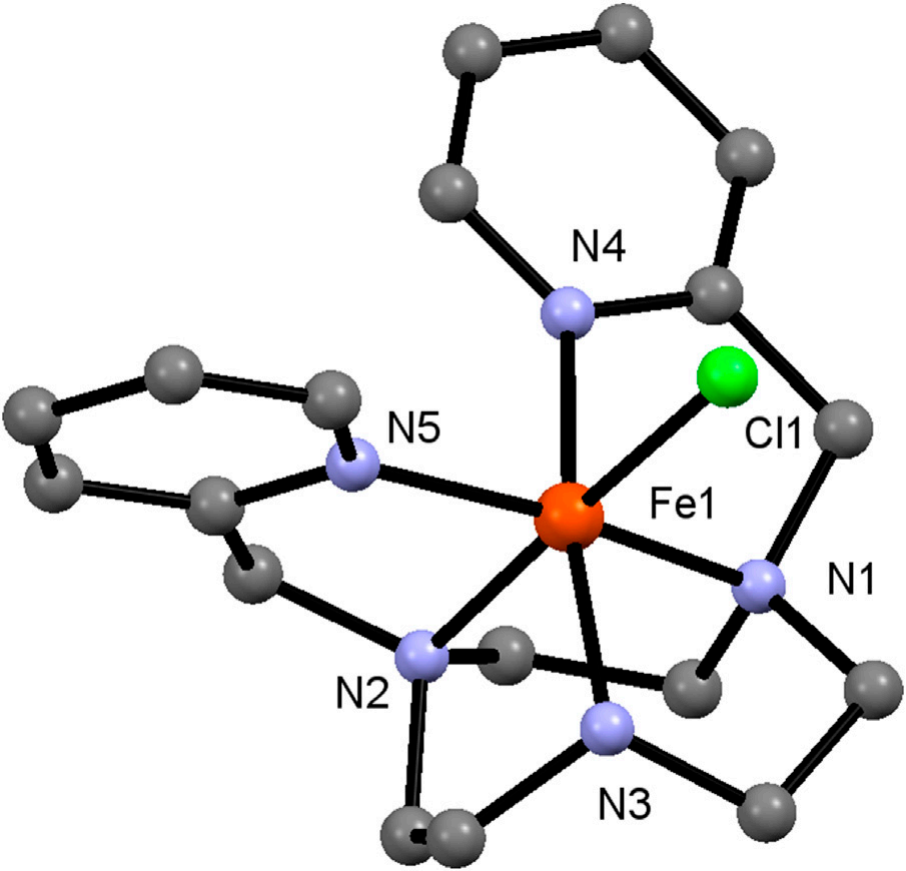
Crystal Structure of **Fe-L** ^**N3**^. Hydrogen atoms and counter anions are omitted for clarity.

The successful complex formation was supported by high resolution MS revealing characteristic peaks at *m/z* 516.0974 (**Fe-L** ^**N3**^), 519.0975 (**Co-L** ^**N3**^), 518.1018 (**Ni-L** ^**N3**^), as well as 533.0584 (**Fe-L** ^**N2S**^), 536.0568 (**Co-L** ^**N2S**^) and 535.0590 (**Ni-L** ^**N2S**^) for the corresponding [**M** + OTf]^+^ fragments (Supplementary Figures S13–S18). To verify the oxidation state of +3 for both the Fe and the Co complexes, different approaches have been made. Since **Co-L** ^**N3**^ and **Co-L** ^**N2S**^ represent diamagnetic compounds, i.e., they are in the low-spin d^6^ form, NMR spectroscopy could be applied. Thereby, **Co-L** ^**N3**^ gives a nicely resolved ^1^H NMR spectrum (Supplementary Figure S11) where all signals can be assigned. The ligand conformation leads to an asymmetry in the complex in contrast to the symmetric nature of the ligand itself (see also crystal structure in Figure 1). Thus, the pyridyl substituents give rise to eight individual signals in the aromatic region, ranging from 9.5 to 7.2 ppm. The methylene groups arise at 5.1, 4.6, and 4.5 ppm and the macrocyclic protons from 4.2 to 2.0 ppm. In case of **Co-L** ^**N2S**^ the ^1^H NMR spectrum shows broad signals in the region from 2 to 9 ppm. It seems that the S-donor atom in the ligand macrocycle is less strongly bound leading to a higher mobility in the system and hence stronger dynamics.

On the other hand, **Fe-L** ^**N3**^ and **Fe-L** ^**N2S**^ are paramagnetic compounds, which give rise to non-interpretable ^1^H NMR spectra. The magnetic properties indicate a +3 oxidation state in octahedral environment with a strong ligand field, as for +2 oxidation state a d^6^ low-spin state, and thus a diamagnetic compound, would be expected. The +3 oxidation state was confirmed by using EPR spectroscopy for both compounds giving a Fe^III^ typical spectrum with signals having a *g* value of around 4.0 and 2.0 (Supplementary Figure S23) (Srinivasan and Gralla, 2002).

Electrochemical and spectroscopic properties were determined *via* cyclic voltammetry (CV) and UV/vis measurements. The UV/vis studies in DMF solution (Table 1; Supplementary Figures S19–S21) reveal a strong ligand-based absorption peaking at 268 nm for all compounds, which can be assigned to be situated at the aromatic picolyl substituents. These maxima are outside the visible spectrum and do not have an impact on the color of the complexes. For the two Fe complexes the dark brown color derives from a broad absorption feature ranging from 600 to 350 nm with a shoulder at around 432 nm (**Fe-L** ^**N3**^ **)** or 502 nm (**Fe-L** ^**N2S**^ **)**. The Ni complex **Ni-L** ^**N3**^ only has an additional absorption maximum at 308 nm and hence is colorless. As the **Ni-L** ^**N2S**^ absorbs in a broader area from 500 to 300 nm it is isolated as light brown solid. The most striking difference is revealed for the Co complexes where d-d transitions are observed at 362 nm (ε = 320 dm^3^/mol*cm) for **Co-L** ^**N3**^ and 490 nm (ε = 390 dm^3^/mol*cm) for **Co-L** ^**N2S**^ leading to a light red/pink color.

**TABLE 1:** Redox potentials [V], excited state redox potentials [V] (marked with *) and MLCT absorption maxima [nm] with corresponding molar attenuation coefficients ɛ [dm^3^ mol^−1^cm^−1^] of the complexes. Peak potentials are given vs. Fc/Fc^+^.

Complex	Irrev. E_red_	revers. E_ox_	λ_(abs)_ (ε)
Fe-L^N3^	−2.50	−0.09	432 (1700)
	−2.91		
Co-L^N3^	−0.64	−0.59	507 (200) ^a^
	−2.17		362 (320) ^a^
	−2.59		
Fe-L^N2S^	−0.34	0.20	502 (790)
	−2.29		
	−2.77		
Co-L^N2S^	−0.71	−0.35	490 (390)
	−1.93		
	−2.43		
	−2.88		
Ir^b^	−2.38 ^c^	+0.91	379 (7,100)
Ir^*^ ^d^	+0.36	−1.84	
Cu^b^	−2.05	+0.93	389 (5100)
Cu*^b^	+0.63	−1.75	

a McLachlan et al. (1995) .

b Giereth et al. (2021) .

c Reversible redox process.

d Teegardin et al. (2016) .

In the CV measurements of the complexes **Fe-L** ^**N3**^ and **Fe-L** ^**N2S**^ a reversible redox event is observed at a half-wave potential of −0.09 and 0.20 V (referenced to Fc/Fc^+^), respectively, representing the Fe^II^/Fe^III^ redox couple (Table 1; Supplementary Figure S22). In addition, there are irreversible reduction events at −2.50 V and −2.91 V for **Fe-L** ^**N3**^ and −2.29 V and −2.77 V for **Fe-L** ^**N2S**^, which can tentatively be assigned to the Fe^II^/Fe^I^ reduction and the formal Fe^I^/Fe^0^ reduction. However, there is certainly the possibility that the true electronic distribution is different and ligand-based reduction cannot be neglected without further experiments.

Interestingly, **Fe-L** ^**N2S**^ is thus easier to reduce (by about 200 mV) than the sulfur free analogue. This result can be attributed to the electron donating property of the sulfur atom, and a similar trend is also observed for the related cobalt complexes. It is important to note, though, that for **Fe-L** ^**N2S**^ an additional reduction process occurs at −0.34 V, which we tentatively relate to the sulfur donor atom. A similar behavior is observed for **Co-L** ^**N2S**^ that shows and additional reduction process at −0.71 V, which is absent for **Co-L** ^**N3**^.

In case of **Co-L** ^**N3**^ a reversible redox event at −0.59 V is indicative for the Co^II^/Co^III^ redox couple. This event is accompanied by two irreversible reduction processes at −2.17 V and −2.59 V resulting in the formation of Co^I^ and finally the Co^0^ state – most likely being a Co^I^(**L** ^**N3** −^) species. In accordance with the observations for **Co-L** ^**N3**^, **Co-L** ^**N2S**^ shows one reversible reduction at −0.35 V and three irreversible reduction events at more negative potentials at −1.93 V, −2.43 V and −2.88 V that are shifted to higher voltages when compared to **Co-L^N3^**. An assignment of these redox processes is not straightforward and currently not possible. The Ni complexes **Ni-L** ^**N3**^ and **Ni-L** ^**N2S**^ do not show any redox processes in the investigated solvent window.

### Crystallography

X-ray quality crystals of **Co-L** ^**N3**^ and **Fe-L** ^**N3**^ were obtained by slow vapor diffusion of Et_2_O to a high concentrated MeCN solution. Both complexes are isostructural (Figure 1; Supplementary Figure S24; Supplementary Tables S1, S2) with all five nitrogen atoms acting as donor atoms. The octahedral coordination sphere is completed by coordination of one chloride ion. The crystal structure shows a hydrogen atom at the secondary amine which confirms the +3 oxidation state (two triflate counter ions were also found per cation).

In case of **Co-L** ^**N3**^ shorter Co-N bond lengths (1.94–1.97 Å) are observed, which is in line with Co^3+^ being smaller than Fe^3+^. The Co-Cl bond distance, on the other hand, is almost identical to the Fe-Cl bond with ∼2.23 Å (Table 2; Supplementary Table S2). For **Co-L** ^**N3**^ an isostructural complex [Co**L^N3^** (OH_2_)][CIO_4_]_3_•H_2_O was published earlier (McLachlan et al., 1995) having similar bond angles and distances, but a shorter Co-O bond since H_2_O is a smaller ligand than the chloride ion. Furthermore, there are a few crystal structures of similar Fe compounds with the **L** ^**N3**^ ligand described in the literature. Two prominent examples are the complexes [Fe**L** ^**N3**^(Cl)](PF_6_) and [Fe**L** ^**N3**^(NCS)](PF_6_), which were obtained *via* reaction under inert conditions and, hence, possess an oxidation state for Fe of +2 (Spiccia et al., 1998). Both complexes nonetheless show a similar ligand geometry, with Fe in an octahedral coordination environment surrounded by five nitrogen and one (pseudo-)halide donor atom.

**TABLE 2:** Selected interatomic distances (Å) and bond angles (°) for Fe-L^N3^.

Fe(1)-Cl(1)	2.223(5)	N(2)-Fe(1)-Cl(1)	174.90(5)
Fe(1)-N(1)	1.972(16)	N(3)-Fe(1)-N(4)	169.67(7)
Fe(1)-N(2)	1.998(15)	N(5)-Fe(1)-N(1)	169.64(6)
Fe(1)-N(3)	1.972(16)	N(2)-Fe(1)-N(5)	82.94(6)
Fe(1)-N(4)	1.997(16)	N(3)-Fe(1)-Cl(1)	90.50(5)
Fe(1)-N(5)	1.974(16)	N(4)-Fe(1)-N(1)	83.37(6)

In case of the **L** ^**N2S**^ complexes we were not able to obtain crystals so far. Nonetheless, crystal structures have been reported for similar Fe^II^ and Ni^II^ complexes (Wasielewski and Mattes, 1993; Zhang et al., 1998).

### Photocatalysis

The photocatalytic activity of the prepared complexes was determined using Ir(dFppy)_3_ and triethylamine (TEA) as sacrificial electron donor. In earlier studies we successfully applied the following conditions (Giereth et al., 2021): 5 × 10^−5^ M catalyst concentration in 5 ml DMF with 5 × 10^−5^ M **Ir** as photosensitizer (PS) and 5 Vol% TEA as sacrificial electron donor (SR). The catalysis mixture is placed in front of a 200 W Hg-lamp equipped with a 400 nm low-wavelength cut-off filter to exclude UV light. **Ir** was selected because of its ability to still absorb solar photons in the region of 400–460 nm (Becker et al., 2020) and its potentially well suited (excited state) redox properties (Table 1) (Koike and Akita, 2014; Lee and Han, 2020; Giereth et al., 2021). For this work we just focused on the gaseous reduction products formed and did not investigate the formation of liquid products. Further information is given in the supplementary data.

**FIGURE 2:**
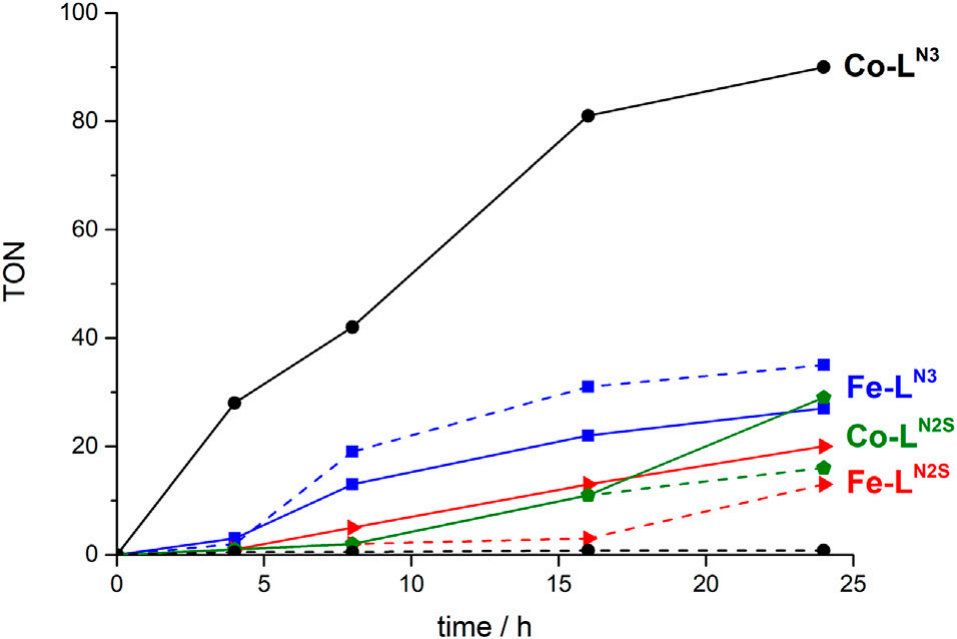
Comparison of catalytic activity of the Fe and Co based catalysts (5 × 10^−5^ M), in DMF applying a λ > 400 nm longpass filter, with 5% TEA as SR, Ir(dFppy)_3_ and a 200 W Hg-lamp. Dotted line: H_2_ formation and solid line: CO formation.

It can be observed, that not only the ligand but also the metal centre do have an impact on both the formed products and the catalytic activity (Figure 2; Table 3). While **Fe-L** ^**N3**^ shows a slightly higher TON for H_2_ than for CO formation after 24 h reaction time, **Co-L** ^**N3**^ shows no H_2_ formation and the highest TON_co_ for the here investigated complexes. This result might be due to the fact that Fe tends to form hydride intermediates (Drosou et al., 2020). When a sulfur atom is added to the ligand framework, considerable H_2_ formation is observed. It is known, that sulfur donor atoms can take part in hydrogen evolution reaction by forming a sulfur-hydride species (Darmon et al., 2014; Drosou et al., 2020). On a side note, the illumination of the reaction solution without any catalysts results in no CO or H_2_ release, indicating that the photosensitizer is stable and its (photo) decomposition products are not responsible for the formation of the gaseous products.

**TABLE 3:** Turn over number (TON) for CO and H_2_ formation for the respective complexes after 24 h of illumination. The catalytic reaction was performed with a catalyst concentration of 5 × 10^-5^ M in DMF with 5% TEA as sacrificial electron donor applying a λ > 400 nm longpass filter and a 200 W Xe-lamp (—no product determined by GC).

Complex	TON_Co_ (24 h)	TON_H2_ (24 h)
Fe-L^N3^	27	35
Co-L^N3^	90	—
Ni-L^N3^	—	—
Fe-L^N2S^	20	13
Co-L^N2s^	29	16
Ni-L^N2S^	—	35

Further evidence for molecular catalysis could be obtained by adding mercury to a catalysis solution with **Co-L** ^**N3**^. Similar TONs were obtained indicating that Co colloids did not form. In case of **Co-L** ^**N2S**^ the ratio of CO:H_2_ changes to about 2:1 (i.e., 66 % selectivity for CO formation) in comparison to no H_2_ formation for **Co-L** ^**N3**^. In case of **Fe-L** ^**N2S**^ the CO:H_2_ ratio stays very similar to the one of **Co-L** ^**N2S**^, but the activity drops by about one third, and about half in comparison to **Fe-L** ^**N3**^. For the Ni complexes the electrochemical data predicted no (photo)catalytic activity, because they do not show a reduction process in the solvent window investigated. This could be confirmed for **Ni-L** ^**N3**^, whereas **Ni-L** ^**N2S**^ shows a moderate TON_H2_, which again is probably induced by the sulfur atom in the ligand system.

In order to investigate if the product ratio or the activity can be adjusted by adding an additional proton source, water was added to a **Co-L** ^**N2S**^ catalytic mixture in different concentrations. Thereby a shift to higher TON_H2_ is observable, while TON_CO_ is decreasing (Supplementary Table S4). When adding 1 Vol.% water a ratio of CO:H_2_ 18:24 was observed, while the addition of 5 Vol.% water led to a ratio of CO:H_2_ 5:54. This result strikingly demonstrates that the product selectivity can be manipulated to a large amount by the addition of a proton source.

To investigate the influence of the type of PS, catalytic experiments were performed using **Cu**. This PS was chosen due to its similar absorption and redox features as compared to **Ir** (Table 1) (Giereth et al., 2021). Unexpectedly, the combination of **Cu** and **Co-L** ^**N3**^ shows only a TON_CO_ of 13 after 24 h of illumination, i.e., only a seventh part compared to the reaction using **Ir**. The much lower overall TON_CO_ can be attributed to the rather fast photodecomposition of **Cu** (Giereth et al., 2021). In the first 2 hours of illumination, **Cu** actually shows a better performance than **Ir** (Supplementary Figure S25; TON_CO_ of 9 *vs.* 3). However, there is almost no further increase in CO evolution for **Cu** indicating that most of the photosensitizer is decomposed already after a few hours of reaction time. The initial better performance of **Cu** *vs.* **Ir** is tentatively assigned to the more suitable excited state oxidation potential of **Cu** (Table 1) and, hence, more efficient oxidative quenching of the excited state by the catalyst (see next section).

### Photophysical Studies

Luminescence quenching experiments were performed to obtain some insight into the reaction mechanism. The excited state of the (Ir) photosensitizer can be quenched oxidatively by the catalyst Co-L^N3^ (Supplementary Figure S26; Table 1). Specifically, Stern-Volmer luminescence quenching experiments in acetonitrile yield a quenching rate constant k_*q*_ = 1.1·10^9^ M^−1^s^−1^ (for Ir), which is roughly a factor of seven (Montalti et al., 2006) below the diffusion limit. Under the catalytically relevant reaction conditions, 5·10^−5^ M Co-L^N3^ was present, and given a rate constant of 1.1·10^9^ M^−1^ s^−1^ for oxidative **Ir** luminescence quenching, this leads to a pseudo first-order rate constant of 5.5·10^4^ s^−1^ for photoinduced electron transfer from **Ir** to **Co-L^N3^**. Given an inherent excited-state decay of 5·10^5^ s^−1^ (lifetime of Ir of about 2 μs) (Giereth et al., 2021) this implies that 11 in 100 photo-excitations (5.5·10^4^ s^−1^/5·10^5^ s^−1^ = 0.11) will lead to electron transfer from **Ir** to **Co-L^N3^** under the catalytically relevant conditions with 5·10^-5^ M **Co-L^N3^**.

Similar bimolecular rate constants were determined by Chan and co-workers (Chan et al., 2015), who investigated the photocatalytic CO_2_ reduction in acetonitrile with [Co^II^(tpa)Cl]Cl (tpa = tris(2-pyridylmethyl)amine) and using Ir(ppy)_3_ (ppy = 2-phenylpyridine anion) as photocatalyst. Stern–Volmer analysis revealed quenching rate constants for the photosensitizer of 5.29 × 10^9^ M^−1^s^−1^ for [Co^II^(tpa)Cl]Cl and 4.14 × 10^4^ M^−1^s^−1^ for TEA, respectively. It was further noted that reductive quenching of excited [Ir(ppy)_3_] is not feasible from thermodynamic consideration (E_1/2_ [(Ir(ppy)_3_)^*^/(Ir(ppy)_3_)^−^] = +0.31 V vs. SCE) and, hence, oxidative quenching of the PS is the more favourable reaction path. These findings are also in line with previous studies by some of us (Giereth et al., 2021), in which we used **Ir** in the presence of a large excess of TEA and a dirhenium catalyst. In the photocatalytic experiments, **Ir** (E_1/2_[(Ir(dFppy)_3_)*/(Ir(dFppy)_3_)^−^] = +0.36 V vs. Fc/Fc^+^) (Teegardin et al., 2016) seemingly had no influence on the catalytic performance, because the dirhenium compound was itself an efficient photocatalyst and because of very inefficient reductive quenching of **Ir** by TEA. It is interesting to note that smaller quantities of a stronger reductant (such as BIH, 1,3-dimethyl-2-phenylbenzimidazoline) gave significantly greater turnover numbers and led to very fast CO_2_ transformation.

Though the fluorinated **Ir** complex is a somewhat stronger photo-oxidant than Ir(ppy)_3_ (+0.36 V vs SCE compared to +0.31 V vs SCE, see above) one may assume that the rate constant for bimolecular quenching of photoexcited **Ir** by TEA is on a similar order of magnitude, roughly 4.14·10^4^ M^−1^s^−1^ (see above). Under the catalytically relevant reaction conditions in which 0.36 M TEA is present, this leads to a pseudo-first order rate constant of 1.5·10^4^ s^−1^. **Co-L** ^**N3**^ is present at 5·10^-5^ M concentration under the catalytically relevant conditions, and given a rate constant of 1.1·10^9^ M^−1^s^−1^ for bimolecular electron transfer from photoexcited **Ir** to **Co-L** ^**N3**^ (see above), one obtains a pseudo-first order rate constant of 5.5·10^4^ s^−1^. Thus, under the assumption that reductive excited-state quenching of **Ir** by TEA is not more rapid than reductive quenching of [Ir(ppy)_3_] despite its 0.05 V higher oxidative power (see above), oxidative quenching of **Ir** by **Co-L** ^**N3**^ is a factor of 3.6 (= 5.5·10^4^ s^−1^/1.5·10^4^ s^−1^) faster than reductive quenching by TEA.

**SCHEME 2:**
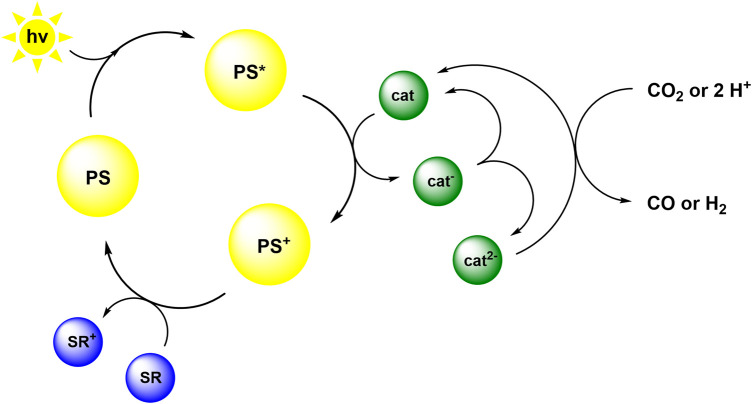
Proposed reaction mechanism based on oxidative quenching of an excited PS and disproportionation of the mono-reduced catalyst.

Therefore we speculate that the reaction mechanism is as illustrated in Scheme 2. Following the absorption of light by the photosensitizer, the excited PS* transfers an electron to the catalyst. Subsequently, PS^+^ is reduced by TEA to reform PS, which then restarts the cycle (Pellegrin and Odobel, 2017). The reduced catalyst cat^−^ is probably only able to interact with protons, derived from TEA decomposition, to form a hydride intermediate. Consecutive electron and proton transfer results in hydrogen formation (Dempsey et al., 2009; Darmon et al., 2014; Wiedner and Bullock, 2016). In order to be able to reduce CO_2_ to CO, the catalyst very likely needs to be present in the double reduced form cat^2−^. The double reduced species could form in a dark reaction by further reduction of cat^−^ by the radical cation TEA^•+^, which is a potent reductant (Shimoda et al., 2018). Alternatively, cat^2−^ could result from a disproportionation reaction (indicated in Scheme 2) as has been proposed for other 3d transition metal compounds (Takeda et al., 2017; Dalle et al., 2019). Such disproportionation reactions have likewise been demonstrated to play a key role in photoinduced charge accumulation (Skaisgirski et al., 2017). In another alternative, the attack of two single reduced catalyst species on CO_2_ could be a possible reaction path, as has been suggested for rhenium complexes (Morris et al., 2009), though this requires the formation of a ternary encounter complex in solution. Further investigations to elucidate the reaction mechanism by characterization and isolation of intermediates are currently performed.

## Experimental

General procedure for the synthesis of complexes **Fe-L** ^**N3**^ and **Co-L** ^**N3**^. Macrocycle **L** ^**N3**^ (1 eq.) was dissolved in MeCN (5 ml) and M(OTf)_2_ (1. eq) was added. After stirring for 3 days at room temperature, the solvent was removed. The resulting oil was washed with CH_2_Cl_2_ and toluene, dissolved in MeCN and precipitated using Et_2_O.

### Synthesis of Fe-L^N3^

After purification **Fe-L** ^**N3**^ was obtained as brownish red solid (38 mg, 54%).

ESI-MS: *m/z:* calc. for [M + OTf^**-**^]^+^, 516.0974; found, 516.0974.

UV/vis: λ 268, 312, 432 nm.

### Synthesis of Co-L^N3^

After purification **Co-L** ^**N3**^ was obtained as pink solid (49 mg, 38%).

^1^H NMR (400 MHz, CD_3_CN): δ 9.24 (d, *J* = 5.8 Hz, 1H), 8.33 (td, *J* = 1.5, 7.8 Hz, 1H), 8.13 (td, *J* = 1.5, 7.8 Hz, 1H_pico_), 7.86 (m, 2H_pico_), 7.72 (d, *J* = 7.9 Hz, 1H_pico_), 7.47 (t, *J* = 7.4, 1H_pico_), 7.21 (d, *J* = 5.9 Hz, 1H_pico_), 6.67 (s, NH), 5.09 (d, *J* = 16.5 Hz, 1H_alkyl_), 4.64 (d, *J* = 16.5 Hz, 1H_alkyl_), 4.46 (m, 2H_alkyl_), 4.10 (m, 2H_macro_), 3.79 (m, 3H_macro_), 3.45 (m, 1H_macro_), 3.31 (dd, *J* = 6.6, 15.2 Hz, 1H_macro_), 3.23 (dd, 5.8, 12.5 Hz, 1H), 3.01 (dd, 5.3, 13.7 Hz, 1H_macro_), 2.77 (td, 5.9, 13.7 Hz, 1H_macro_), 2.54 (dd, 5.9, 13.7 Hz, 1H_macro_), 2.01(m, 1H_macro_).^13^C NMR (400 MHz, CD_3_CN): δ 164.83 (C_quart_), 162.48 (C_quart_), 153.65 (C_pico_), 149.45 (C_pico_), 141.83 (C_Pico_), 140.83 (C_Pico_), 126.69 (C_Pico_), 126.61 (C_Pico_), 125.51 (C_Pico_), 129.94 (C_Pico_), 69.43 (C_alkyl_), 67.27 (C_alkyl_), 63.92 (C_macro_), 62.37 (C_macro_), 61.53 (C_macro_), 60.16 (C_macro_), 54.23 (C_macro_), 52.93 (C_macro_). ^19^F NMR (300 MHz, CD_3_CN): δ -76.95 (F_triflat_).

ESI-MS: *m/z*: calc. for [M + OTf^**-**^]^+^, 519.0956; found, 519.0975.

UV/Vis: 268, 362, 507 nm.

General procedure for the synthesis of **Ni-L** ^**N3**^, **Fe-L** ^**N2S**^, **Co-L** ^**N2S**^ and **Ni-L** ^**N2S**^. The macrocycles **L** ^**N3**^ and **L** ^**N2S**^ were dissolved in dry MeCN (5 ml) and M(OTf)_2_ (1 eq.) was added. The suspension was stirred for 3 days at room temperature and the solvent was removed subsequently. The resulting oil was washed with CH_2_Cl_2_ and toluene [Fe(OTf)_2_ and Co(OTf)_2_] or THF [Ni(OTf)_2_], dissolved in MeCN and the remaining chloride ions were precipitated using an excess of Ag(OTf). The filtrate was evaporated and afterwards the residue was taken up in a small amount of MeCN and precipitated using Et_2_O.

### Synthesis of Ni-L^N3^

After purification **Ni-L** ^**N3**^ was obtained as beige solid (39 mg, 30%).

ESI-MS: *m/z*: calc. for [M + OTf^**-**^]^+^, 518.0984; found, 518.1018.

UV/Vis: 268, 308 nm.

### Synthesis of Fe-L^N2S^

After purification **Fe-L** ^**N2S**^ was obtained as dark brown solid (34 mg, 46%).

ESI-MS: *m/z*: calc. for [M + OTf^**-**^]^+^, 533.0586; found, 533.0584.

UV/vis: 268, 490, 502 nm.

### Synthesis of Co-L^N2S^

After purification **Co-L** ^**N2S**^ was obtained as red solid (31 mg, 50%).

ESI-MS: *m/z*: calc. for [M + OTf^**-**^]^+^, 536.0571; found, 536.0568.

UV/vis: 268, 490 nm.

### Synthesis of Ni-L^N2S^

After purification **Ni-L** ^**N2S**^ was obtained as light brown solid (28 mg, 44%).

ESI-MS: *m/z*: calc. for [M + OTf^**-**^]^+^, 535.0590; found, 535.0590.

UV/vis: 268 nm.

## Conclusion

We herein describe the synthesis of two series of macrocyclic complexes **M-L** ^**N3**^ and **M-L** ^**N2S**^ containing Fe, Co and Ni, which can be applied in the photocatalytic activation of CO_2_. Initially, an Ir photosensitizer was used and the combination of **Ir** and **Co-L** ^**N3**^ showed the highest TON_CO_ (90 after 24 h of illumination) of all investigated complexes. The ratio of the gaseous products CO and H_2_ can be varied by the choice of metal ion, macrocyclic ligand and solvent composition. The product selectivity can even be adjusted to almost solely formation of CO (in case of **Co-L** ^**N3**^) or H_2_ (in case of **Ni-L** ^**N2S**^). In addition, we were able to demonstrate that a Cu photosensitizer can be used for the catalytic reaction as well, making the whole system solely 3d metal based. The initially higher catalytic activity compared to **Ir** (TON_CO_ of 9 vs. 3 after 2 h reaction time) can tentatively be assigned to the more positive excited state oxidation potential of **Cu** making the oxidative quenching process by the catalyst more efficient. On the contrary, the long-term stability of the Cu photosensitizer is clearly inferior compared to the Ir photosensitizer with a TON_CO_ after 24 h of illumination of 13 vs. 90.

First mechanistic investigations confirm that an oxidative quenching mechanism is more likely than a reductive quenching process. Further experiments are on the way to confirm possible intermediates involved, such as a hydride species, and the influence of further reaction parameters on the outcome of the photocatalytic reaction, such as changing the sacrificial electron donor. The latter might also influence the quenching process and thus the overall TON and product selectivity.

## Data Availability

The raw data supporting the conclusion of this article are included in the article/Supplementary Material, further inquiries can be directed to the corresponding author.

## References

[B1] Aresta M. (2010). Carbon Dioxide as Chemical Feedstock. Weinheim, Germany: Wiley‐Vch Verlag Gmbh & Co. Kgaa.

[B2] Becker M. R. Wearing E. R. Schindler C. S. (2020). Synthesis of Azetidines via Visible-Light-Mediated Intermolecular [2+2] Photocycloadditions. Nat. Chem. 12 (10), 898–905. 10.1038/s41557-020-0541-1 32968230

[B3] Benson E. E. Kubiak C. P. Sathrum A. J. Smieja J. M. (2009). Electrocatalytic and Homogeneous Approaches to Conversion of CO_2_ to Liquid Fuels. Chem. Soc. Rev. 38 (1), 89–99. 10.1039/b804323j 19088968

[B4] Benvenuti M. Meneghello M. Guendon C. Jacq-Bailly A. Jeoung J.-H. Dobbek H. (2020). The Two CO-dehydrogenases of Thermococcus Sp. AM4. Biochim. Biophys. Acta (Bba) - Bioenerg. 1861 (7), 148188. 10.1016/j.bbabio.2020.148188 32209322

[B5] Cao R. Müller P. Lippard S. J. (2010). Tripodal Tris-Tacn and Tris-Dpa Platforms for Assembling Phosphate-Templated Trimetallic Centers. J. Am. Chem. Soc. 132 (49), 17366–17369. 10.1021/ja108212v 21090678

[B6] Chak B. McAuley A. Whitcombe T. W. (1994). Crystal and Solution Structure of a Pendant-Armed Macrocyclic Complex of Palladium(II). Can. J. Chem. 72 (6), 1525–1532. 10.1139/v94-189

[B7] Chan S. L.-F. Lam T. L. Yang C. Yan S.-C. Cheng N. M. (2015). A Robust and Efficient Cobalt Molecular Catalyst for CO_2_ Reduction. Chem. Commun. 51 (37), 7799–7801. 10.1039/c5cc00566c 25783610

[B8] Chen L. Chen G. Leung C.-F. Yiu S.-M. Ko C.-C. Anxolabéhère-Mallart E. (2014). Dual Homogeneous and Heterogeneous Pathways in Photo- and Electrocatalytic Hydrogen Evolution with Nickel(II) Catalysts Bearing Tetradentate Macrocyclic Ligands. ACS Catal. 5 (1), 356–364. 10.1021/cs501534h

[B9] Dalle K. E. Warnan J. Leung J. J. Reuillard B. Karmel I. S. Reisner E. (2019). Electro- and Solar-Driven Fuel Synthesis with First Row Transition Metal Complexes. Chem. Rev. 119 (4), 2752–2875. 10.1021/acs.chemrev.8b00392 30767519 PMC6396143

[B10] Darmon J. M. Raugei S. Liu T. Hulley E. B. Weiss C. J. Bullock R. M. (2014). Iron Complexes for the Electrocatalytic Oxidation of Hydrogen: Tuning Primary and Secondary Coordination Spheres. ACS Catal. 4 (4), 1246–1260. 10.1021/cs500290w

[B11] Dempsey J. L. Brunschwig B. S. Winkler J. R. Gray H. B. (2009). Hydrogen Evolution Catalyzed by Cobaloximes. Acc. Chem. Res. 42 (12), 1995–2004. 10.1021/ar900253e 19928840

[B12] Drosou M. Kamatsos F. Mitsopoulou C. A. (2020). Recent Advances in the Mechanisms of the Hydrogen Evolution Reaction by Non-innocent Sulfur-Coordinating Metal Complexes. Inorg. Chem. Front. 7 (1), 37–71. 10.1039/c9qi01113g

[B13] Esselborn J. Muraki N. Klein K. Engelbrecht V. Metzler-Nolte N. Apfel U.-P. (2016). A Structural View of Synthetic Cofactor Integration into [FeFe]-Hydrogenases. Chem. Sci. 7 (2), 959–968. 10.1039/c5sc03397g 29896366 PMC5954619

[B14] Francke R. Schille B. Roemelt M. (2018). Homogeneously Catalyzed Electroreduction of Carbon Dioxide-Methods, Mechanisms, and Catalysts. Chem. Rev. 118 (9), 4631–4701. 10.1021/acs.chemrev.7b00459 29319300

[B15] Friscourt F. Fahrni C. J. Boons G.-J. (2012). A Fluorogenic Probe for the Catalyst-free Detection of Azide-Tagged Molecules. J. Am. Chem. Soc. 134 (45), 18809–18815. 10.1021/ja309000s 23095037 PMC3525324

[B16] Gerschel P. Warm K. Farquhar E. R. Englert U. Reback M. L. Siegmund D. (2019). Apfel, U. P.Sulfur Substitution in a Ni(cyclam) Derivative Results in Lower Overpotential for CO_2_ Reduction and Enhanced Proton Reduction. Dalton Trans. 48 (18), 5923–5932. 10.1039/c8dt04740e 30624449 PMC6504587

[B17] Ghosh D. Sinhababu S. Santarsiero B. D. Mankad N. P. (2020). A W/Cu Synthetic Model for the Mo/Cu Cofactor of Aerobic CODH Indicates that Biochemical CO Oxidation Requires a Frustrated Lewis Acid/Base Pair. J. Am. Chem. Soc. 142 (29), 12635–12642. 10.1021/jacs.0c03343 32598845 PMC9307224

[B18] Giereth R. Obermeier M. Forschner L. Karnahl M. Schwalbe M. Tschierlei S. (2021). Exploring the Full Potential of Photocatalytic Carbon Dioxide Reduction Using a Dinuclear Re_2_Cl_2_ Complex Assisted by Various Photosensitizers. ChemPhotoChem 5, 644–653. 10.1002/cptc.202100034

[B19] Gotico P. Del Vecchio A. Audisio D. Quaranta A. Halime Z. Leibl W. (2018). Visible-Light-Driven Reduction of CO_2_ to CO and its Subsequent Valorization in Carbonylation Chemistry and ^13^C Isotope Labeling. ChemPhotoChem 2 (8), 715–719. 10.1002/cptc.201800012

[B20] Guo Z. Cheng S. Cometto C. Anxolabéhère-Mallart E. Ng S.-M. Ko C.-C. (2016). Highly Efficient and Selective Photocatalytic CO_2_ Reduction by Iron and Cobalt Quaterpyridine Complexes. J. Am. Chem. Soc. 138 (30), 9413–9416. 10.1021/jacs.6b06002 27443679

[B21] Hong D. Kawanishi T. Tsukakoshi Y. Kotani H. Ishizuka T. Kojima T. (2019). Efficient Photocatalytic CO_2_ Reduction by a Ni(II) Complex Having Pyridine Pendants through Capturing a Mg^2+^ Ion as a Lewis-Acid Cocatalyst. J. Am. Chem. Soc. 141 (51), 20309–20317. 10.1021/jacs.9b10597 31726829

[B22] Hong D. Tsukakoshi Y. Kotani H. Ishizuka T. Kojima T. (2017). Visible-Light-Driven Photocatalytic CO_2_ Reduction by a Ni(II) Complex Bearing a Bioinspired Tetradentate Ligand for Selective CO Production. J. Am. Chem. Soc. 139 (19), 6538–6541. 10.1021/jacs.7b01956 28453267

[B23] Iffland L. Siegmund D. Apfel U. P. (2020). Electrochemical CO_2_ and Proton Reduction by a Co(dithiacyclam) Complex. Z. Anorg. Allg. Chem. 646 (13), 746–753. 10.1002/zaac.201900356

[B24] Koike T. Akita M. (2014). Visible-light Radical Reaction Designed by Ru- and Ir-Based Photoredox Catalysis. Inorg. Chem. Front. 1 (8), 562–576. 10.1039/c4qi00053f

[B25] Kumar B. Brian J. P. Atla V. Kumari S. Bertram K. A. White R. T. (2016). New Trends in the Development of Heterogeneous Catalysts for Electrochemical CO_2_ Reduction. Catal. Today 270, 19–30. 10.1016/j.cattod.2016.02.006

[B26] Lee S. Han W.-S. (2020). Cyclometalated Ir(III) Complexes towards Blue-Emissive Dopant for Organic Light-Emitting Diodes: Fundamentals of Photophysics and Designing Strategies. Inorg. Chem. Front. 7 (12), 2396–2422. 10.1039/d0qi00001a

[B27] Liu D.-C. Wang H.-J. Wang J.-W. Zhong D.-C. Jiang L. Lu T.-B. (2018). Highly Efficient and Selective Visible-Light Driven CO_2_-to-CO Conversion by a Co-based Cryptate in H_2_O/CH_3_CN Solution. Chem. Commun. 54 (80), 11308–11311. 10.1039/c8cc04892d 30234862

[B28] Lubitz W. Ogata H. Rüdiger O. Reijerse E. (2014). Hydrogenases. Chem. Rev. 114 (8), 4081–4148. 10.1021/cr4005814 24655035

[B52] Luk’yanenko N. G. Basok S. S. Filonova L. K. Kulikov N. V. Pastushok V. N. (1990). Macroheterocycles. 51. Synthesis of Macrocyclic Polyamines in a Biphasic System. Chem. Heterocycl. Comp. 26, 346–349. 10.1007/BF00472559

[B29] Ma J. Sun N. Zhang X. Zhao N. Xiao F. Wei W. (2009). A Short Review of Catalysis for CO_2_ Conversion. Catal. Today 148 (3-4), 221–231. 10.1016/j.cattod.2009.08.015

[B30] McLachlan G. A. Brudenell S. J. Fallon G. D. Martin R. L. Spiccia L. Tiekink E. R. T. (1995). Synthesis, Structure and Properties of Cobalt(III) Complexes of Pentadentate Ligands with Pyridyl Pendant Arms. J. Chem. Soc. Dalton Trans. 1995 (1), 439–447. 10.1039/DT9950000439

[B31] Montalti M. Credi A. Prodi L. Gandolfi M. T. (2006). Handbook of Photochemistry. 3rd Edition. Boca Raton, Florida: CRC Press, 664. 10.1201/9781420015195

[B32] Morris A. J. Meyer G. J. Fujita E. (2009). Molecular Approaches to the Photocatalytic Reduction of Carbon Dioxide for Solar Fuels. Acc. Chem. Res. 42 (12), 1983–1994. 10.1021/ar9001679 19928829

[B33] Pellegrin Y. Odobel F. (2017). Sacrificial Electron Donor Reagents for Solar Fuel Production. Comptes Rendus Chim. 20 (3), 283–295. 10.1016/j.crci.2015.11.026

[B34] Ringsmuth A. K. Landsberg M. J. Hankamer B. (2016). Can Photosynthesis Enable a Global Transition from Fossil Fuels to Solar Fuels, to Mitigate Climate Change and Fuel-Supply Limitations? Renew. Sustain. Energ. Rev. 62, 134–163. 10.1016/j.rser.2016.04.016

[B35] Schrag D. P. (2007). Preparing to Capture Carbon. Science 315 (5813), 812–813. 10.1126/science.1137632 17289991

[B36] Shimoda T. Morishima T. Kodama K. Hirose T. Polyansky D. E. Manbeck G. F. (2018). Photocatalytic CO_2_ Reduction by Trigonal-Bipyramidal Cobalt(II) Polypyridyl Complexes: The Nature of Cobalt(I) and Cobalt(0) Complexes upon Their Reactions with CO_2_, CO, or Proton. Inorg. Chem. 57 (9), 5486–5498. 10.1021/acs.inorgchem.8b00433 29696969

[B37] Skaisgirski M. Guo X. Wenger O. S. (2017). Electron Accumulation on Naphthalene Diimide Photosensitized by [Ru(2,2′-Bipyridine)_3_]^2+^ . Inorg. Chem. 56 (5), 2432–2439. 10.1021/acs.inorgchem.6b02446 28230991

[B38] Sönnchen N. (2020). BP Energy OutlookBP Energy Outlook 2040 - Summary Tables.

[B39] Spiccia L. Fallon G. D. Grannas M. J. Nichols P. J. Tiekink E. R. T. (1998). Synthesis and Characterisation of Mononuclear and Binuclear Iron(II) Complexes of Pentadentate and Bis(pentadentate) Ligands Derived from 1,4,7-triazacyclononane. Inorg. Chim. Acta 279, 192–199. 10.1016/S0020-1693(98)00122-4

[B40] Srinivasan C. Butler Gralla E. (2002). Measurement of "Free" or Electron Paramagnetic Resonance-Detectable Iron in Whole Yeast Cells as Indicator of Superoxide Stress. Method. Enzymol. 349, 173–180. 10.1016/S0076-6879(02)49333-0 11912907

[B41] Stavila V. Allali M. Canaple L. Stortz Y. Franc C. Maurin P. (2008). Significant Relaxivity gap between a Low-Spin and a High-Spin Iron(ii) Complex of Structural Similarity: an Attractive Off-On System for the Potential Design of Responsive MRI Probes. New J. Chem. 32 (3), 428–435. 10.1039/b715254j

[B42] Takeda H. Cometto C. Ishitani O. Robert M. (2017). Electrons, Photons, Protons and Earth-Abundant Metal Complexes for Molecular Catalysis of CO_2_ Reduction. ACS Catal. 7 (1), 70–88. 10.1021/acscatal.6b02181

[B43] Tamaki Y. Ishitani O. (2017). Supramolecular Photocatalysts for the Reduction of CO_2_ . ACS Catal. 7 (5), 3394–3409. 10.1021/acscatal.7b00440

[B44] Tamaki Y. Koike K. Ishitani O. (2015). Highly Efficient, Selective, and Durable Photocatalytic System for CO_2_ Reduction to Formic Acid. Chem. Sci. 6 (12), 7213–7221. 10.1039/c5sc02018b 29861957 PMC5947534

[B45] Tamaki Y. Koike K. Morimoto T. Ishitani O. (2013). Substantial Improvement in the Efficiency and Durability of a Photocatalyst for Carbon Dioxide Reduction Using a Benzoimidazole Derivative as an Electron Donor. J. Catal. 304, 22–28. 10.1016/j.jcat.2013.04.002

[B46] Teegardin K. Day J. I. Chan J. Weaver J. (2016). Advances in Photocatalysis: A Microreview of Visible Light Mediated Ruthenium and Iridium Catalyzed Organic Transformations. Org. Process. Res. Dev. 20 (7), 1156–1163. 10.1021/acs.oprd.6b00101 27499607 PMC4972501

[B47] Thoi V. S. Kornienko N. Margarit C. G. Yang P. Chang C. J. (2013). Visible-light Photoredox Catalysis: Selective Reduction of Carbon Dioxide to Carbon Monoxide by a Nickel N-Heterocyclic Carbene-Isoquinoline Complex. J. Am. Chem. Soc. 135 (38), 14413–14424. 10.1021/ja4074003 24033186

[B48] Wasielewski K. Mattes R. (1993). Nickel-, Palladium- und Platinkomplexe funktionalisierter Makrocyclen. Die Kristallstrukturen von [Ni(py_2_-tasn)(H_2_O)](ClO_4_)_2_, [Pd(py_2_-tasn)](PF_6_)_2_ und [Pt(py_2_-tasn)](PF_6_)_2_. (py_2_-tasn=4,7-Bis(2-methylpyridyl)-1-thia-4,7-diazacyclononan. Z. Anorg. Allg. Chem. 619, 158–162. 10.1002/zaac.19936190126

[B49] Wiedner E. S. Bullock R. M. (2016). Electrochemical Detection of Transient Cobalt Hydride Intermediates of Electrocatalytic Hydrogen Production. J. Am. Chem. Soc. 138 (26), 8309–8318. 10.1021/jacs.6b04779 27300721

[B50] Wilson J. (2007). Synthesis of Biologically Active Heterocyclic Compounds. Great Britain: University of Glasgow. Available at: http://theses.gla.ac.uk/45/ .

[B51] Zhang D. Busch D. H. Alcock N. W. (1998). Synthesis, Characterization, and Crystal Structures of Iron(II) and Manganese(II) Complexes with 4,7-Bis(2-Pyridylmethyl)-1-Thia-4,7-Diazacyclononane. Bull. Korean Chem. Soc. 10 (9), 897–906.

