# Supplementary material for: Sensitized Photocatalytic CO2 Reduction With Earth Abundant 3d Metal Complexes Possessing Dipicolyl-Triazacyclononane Derivatives
Source: Front Chem. 2021 Sep 30;9:751716. doi: 10.3389/fchem.2021.751716 (PMC8514774; doi:10.3389/fchem.2021.751716)
Supplement: Supplementary file 1 [file DataSheet1.pdf]

frontiers

Supplementary Material

Table of Data

1. **Experimental Details** **page 1**
2. **Synthetic Details** **page 3**
3. **MS spectra** **page 17**
4. **UV/vis measurements** **page 23**
5. **Cyclovoltammetry** **page 25**
6. **EPR spectroscopy** **page 26**
7. **Crystallographic data** **page 27**
8. **Photocatalytical and photophysical studies** **page 29**

1 Experimental Details

NMR spectra were recorded on a Bruker Avance I 400 or Avance II 300 at 293 K (unless noted otherwise) and processed with the software MestReNova (Version 9.0). The chemical shifts δ are reported in parts per million (ppm). ^{1}H and ^{13}C NMR shifts are referenced according to the applied deuterated solvent as internal standard. Coupling constants J are presented as absolute values in Hz, without considering the kind of the coupling. For the characterization of the NMR signals the following abbreviations are used: s = singlet, d = doublet, t = triplet, q = quartet, m = multiplet, dd = doublet of doublets and br = broad. Signal assignment was possible by using different 2D NMR techniques, such as COSY, HSQC, HMBC and NOESY. All of these experiments were measured using standard Bruker pulse sequences and parameters.

Mass spectrometry. High resolution mass spectra were measured using electrospray ionization (ESI) on a Thermo Finnigan LTQ FT instrument or an Agilent Technologies 6210 TOF in the positive as well as negative mode. MS values are given as *m/z*. **M** is defined as $[M\text{-}L^{N3}]^{2+}$ or $[M\text{-}L^{N2S}]^{2+}$ with (M = Fe, Co, Ni). The Fe^{III} and Co^{III} compounds are reduced during the ionization process to the +2 oxidation states (with chloride dissociation – if present). Thus, for all compounds the $[\mathbf{M}+OTf^{-}]^{+}$ ion is detected.

UV/vis measurements. The UV/vis measurements were performed with a Varian Cary 100 spectrophotometer.

Cyclic voltammetry was carried out in dimethylformamide with 0.1 M Bu_4NPF_6 as the supporting electrolyte. The measurements were performed with an Autolab potentiostat PGSTAT101 from Metrohm using a three-electrode configuration. As working electrode, a glassy carbon disc with a 3 mm diameter stick was used. The counter electrode was a Pt electrode. As reference electrode a non-aqueous Ag/Ag^{+} electrode (0.01 M $AgNO_3$ in acetonitrile) was utilized with the ferrocene/ferrocinium (Fc/Fc^{+}) couple as reference, added to the solution after each measurement. Thus, all reported potentials are *versus* the Fc/Fc^{+} couple.

X-ray diffraction. The data collections were performed with a BRUKER D8 VENTURE area detector with Mo-Kα radiation (λ = 0.71073 Å). Multi-scan absorption corrections implemented in SADABS[1] were applied to the data. The structures were solved by intrinsic phasing method (SHELXT-2014)[2] and refined by full matrix least square procedures based on F^2 with all measured reflections (SHELXL-2018)[3] with anisotropic temperature factors for all non-hydrogen atoms. All hydrogen atoms were added geometrically and refined by using a riding model. Crystals for both complexes **Fe-L^{N3}** and **Co-L^{N3}** were obtained by slow vapor diffusion of Et_2O to a high concentrated solution in MeCN. CCDC numbers 2096076 and 2096077 contain the supplementary crystallographic data for this paper. These data can be obtained free of charge from The Cambridge Crystallographic Data Centre via www.ccdc.cam.ac.uk/data_request/cif.

EPR spectra were recorded on a Bruker EMXplus X-band EPR spectrometer and a Bruker-ColdEdge-ER4112HV-CF10-H, Helium recirculating cryostat. The sample solution in the quartz EPR tube was frozen in liquid nitrogen and kept frozen until measured.

Photocatalysis. A LOT-QuantumDesign GmbH 200 W Hg lamp was used for photocatalytic CO_2 reduction experiments. The λ>400 nm longpass filter used was sourced from LOT-QuantumDesign GmbH with the purpose of allowing only the light at greater wavelengths of the employed longpass filter to be transmitted. Each longpass filter is dielectrically coated and has an edge slope of 2%. The concentration dependent and time dependent measurements involving dry TEA (triethylamine) as the electron donor, 4.75 mL of a stock solution of the catalyst in dry DMF and 0.25 mL of dry TEA were added to a Schlenk vessel under inert conditions. Afterwards an equivalent amount of external photosensitizer stock solution was added under inert conditions. Schlenk vessels were employed that could host 5 mL of solution and a further 11 ml space for gas. In the case of water addition, a corrected headspace volume was used for the calculation of the TON. The solution was degassed thoroughly with CO_2 for at least ten minutes and then the vessel sealed. Using a gas tight syringe, 250 µL of the gas phase was injected into the gas chromatograph (GC) and the TON_{CO} or TON_{H2} were determined by taking the area of the peak corresponding to CO (with a retention time of 2.8-3.1 min^{-1}) respectively H_2 (with a retention time of 0.4-0.6 min^{-1}) and corroboration of this value with the calibration curve to identify the TON_{CO} or TON_{H2} value. The Shimadzu GC-2014 gas chromatograph was used for CO or H_2 detection with a thermal conductivity detector and a Resteks ShinCarbon packed column ST 80/100 (2 m, 1/8" outer diameter, 2 mm inner diameter). The injector temperature was set to 200°C, the detector temperature set to 300°C and the gases were separated according to a temperature-time program on the column. CO and persistent gases such as argon and nitrogen were separated at the early stages of the measurement at an oven temperature of 40°C and after 20 minutes CO_2 was eluted at the later stages. It was tried to determine the amount of formic acid which might have been formed, but no NMR method could be found. This is due to the combination of small amounts of formic acid being formed and the used solvent DMF having a signal close to the one of formic acid, hence overlapping that signal occasionally.

2 Synthetic Details

All reactions were performed under a dry Ar or N_2 atmosphere using standard Schlenk techniques or by working in a glovebox. Starting materials and chemicals were obtained from commercial suppliers and used without further purification. The photosensitizer Ir(dFppy)$_3$ was obtained from commercial supplier (Sigma-Aldrich), while the photosensitizer [Cu(xant)(bcp)]$^+$ was provided by the group of Dr. Michael Karnahl (TU Braunschweig, Germany). All solvents were dried and degassed according to standard methods or directly taken from MBraun solvent purification system (e.g. Et_2O or MeCN). Dry DMF (99.8%) was purchased from Sigma-Aldrich and stored over molecular sieve (3 Å) prior to use. Thin-layer chromatography was performed using Merck TLC neutral aluminum oxide or silica gel 60 F254 sheets. For column chromatography, silica gel with a pore size of 60 Å from Acros Organics or neutral AlOx gel was used. The used solvent mixtures are given by volume fractions.

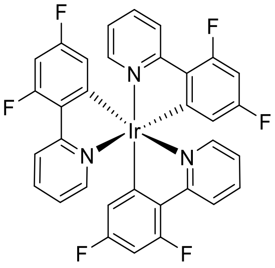

Ir(dFppy)$_3$

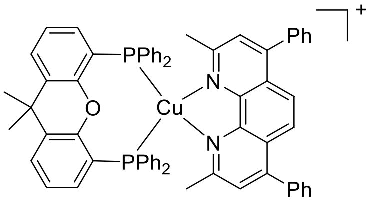

[Cu(xant)(bcp)]$^+$

2.1 Synthesis of ligands

The synthesis of the ligands **L^{N3} (4)** and **L^{N2S} (9)** are outlined in Scheme S1 and Scheme S2. Di-tosyl-ethylenglykol and tri-tosyl-ethylentriamine were synthesized according to published procedures.[4]

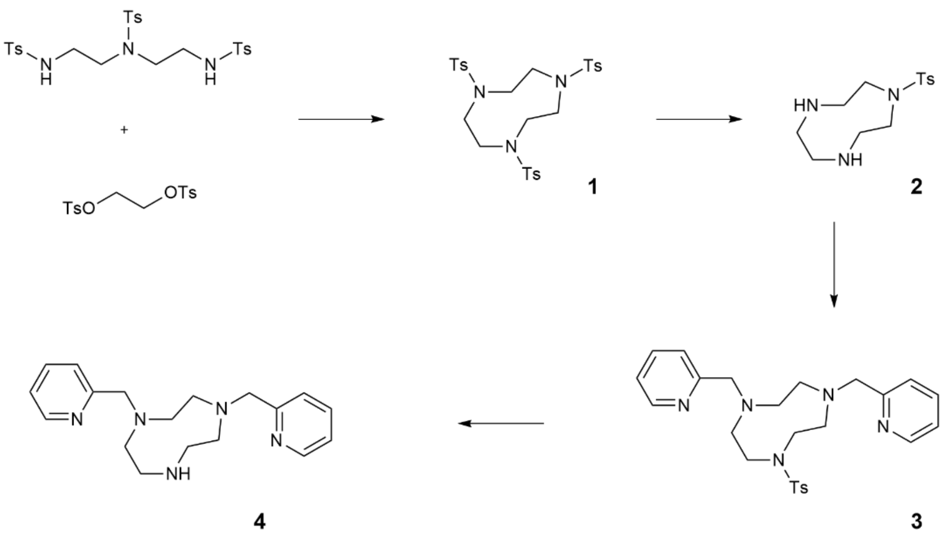

Scheme S1: Synthetic pathway of **L^{N3}**.

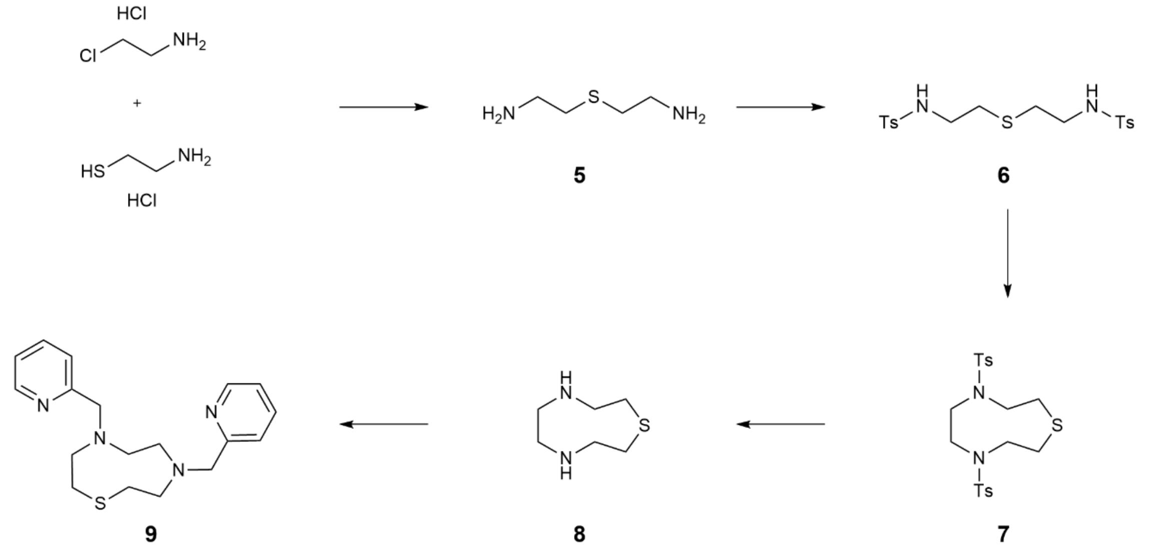

Scheme S2: Synthetic pathway of **L^{N2S}**.

2.1.1 Synthesis of N,N',N"-tri-tosyl-1,4,7-triazacyclononane (1)

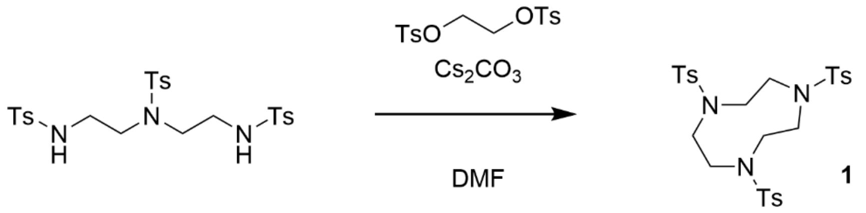

A mixture of Cs_2CO_3 (9.12 g, 28.00 mmol, 2.15 eq.) and tri-tosyl-ethylentriamine (7.35 g, 13.00 mmol, 1 eq.) were dried under vacuum at 60 °C for 1 h. The mixture was suspended using dry DMF (80 mL) and vigorously stirred for 1.5 h. Di-tosyl-ethylene glycol (4.82 mg, 13.00 mmol, 1 eq.) dissolved in another 50 mL of dry DMF were then added over 3 h using a syringe pump, and stirred for 3 days at room temperature. The resulting suspension was slowly poured into 400 mL of water with stirring giving a white precipitate. The solid was filtered, washed with water and recrystallized in 200 mL of 1:1 (v:v) DMF-H_2O. The remaining solid was filtered and dissolved in sufficient CH_2Cl_2. The organic phase was washed with water and the desired product was precipitated with addition of cold EtOH (100 mL). The resulting colorless crystals of pure **1** were filtered and dried under vacuum (6.44 g, 84%).[5]

^{1}H NMR (300 MHz, $CDCl_3$): δ 7.70 (d, J = 8.3 Hz, 4H), 7.32 (d, *J* = 8.3 Hz, 4H), 3.42 (s, 12H), 2.43 (s, 9H).

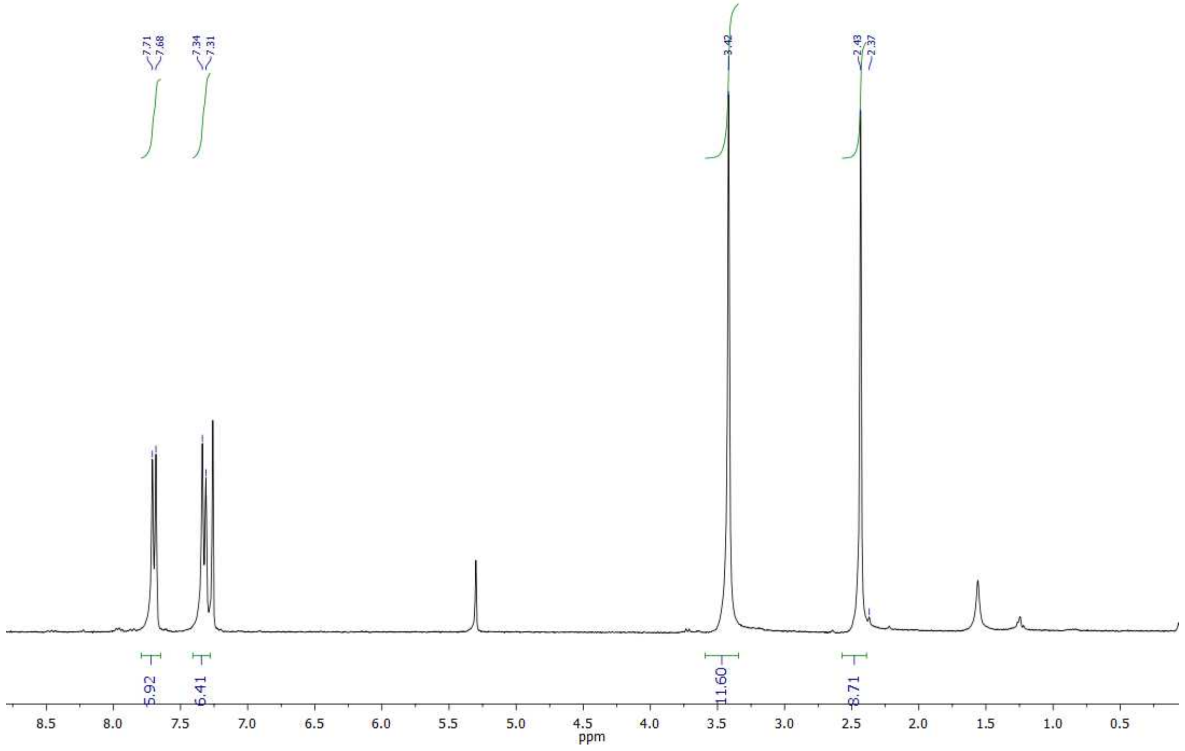

Figure S1: ^{1}H NMR spectrum (300 MHz) of **1** in $CDCl_3$.

2.1.2 Synthesis of 1-tosyl-1,4,7-triazacyclononane (2)

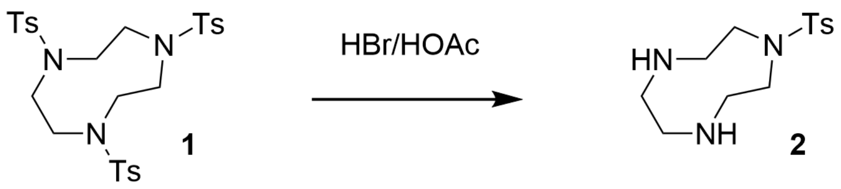

1 (3.10 g, 5.24 mmol, 1 eq.) was combined with PhOH (3.70 g, 39.31 mmol, 7.5 eq.) and dissolved in HBr in glacial acetic acid (33%, 50 mL). The mixture was stirred for 36 h at 90 °C. The resulting solid was filtered, washed with Et_2O and dissolved in sufficient 1 M NaOH. The pink solution was extracted using a large quantity of $CHCl_3$ and the solvent was thereafter removed. **2** was obtained as colorless crystalline powder (1.30 g, 88%).[6]

^{1}H NMR (300 MHz, $CDCl_3$): δ 7.69 (d, J = 8.1 Hz, 2H), 7.31 (d, J = 8.1 Hz, 2H), 3.22 – 3.15 (m, 4H), 3.12 – 3.15 (m, 4H), 2.89 (s, 4H), 1.32 (s, 3H).

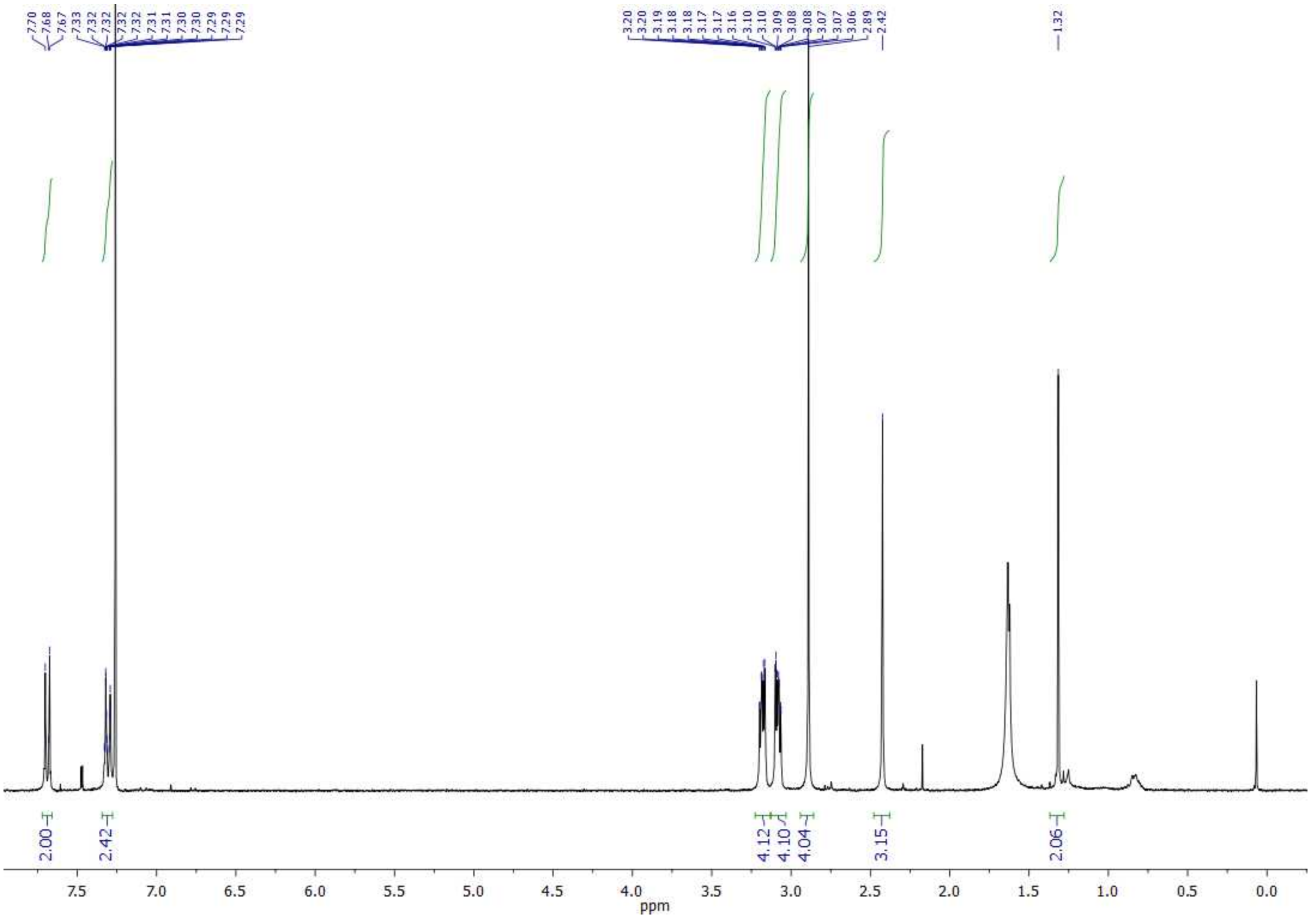

Figure S2: ^{1}H NMR spectrum (300 MHz) of **2** in $CDCl_3$.

2.1.3 Synthesis of 1-tosyl-4,7-di(picolyl)-1,4,7-triazacyclononane (3)

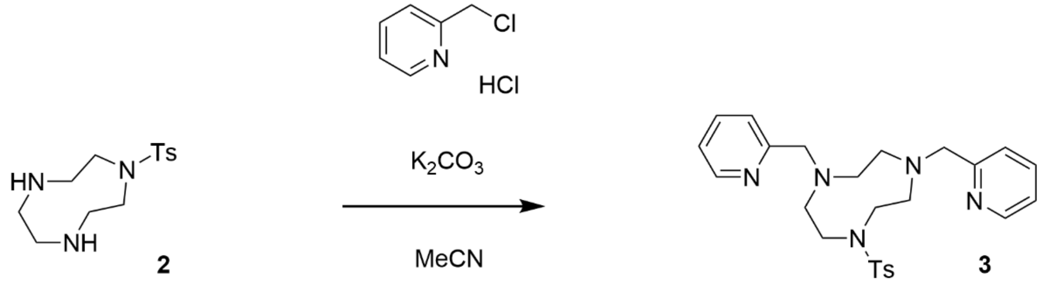

2 (1.02 g, 3.60 mmol, 1 eq.) was dissolved in dry MeCN (50 mL) under inert conditions, K_2CO_3 (4.00 g, 29.00 mmol, 8 eq.) was added and the suspension was stirred for 1 h at room temperature. 2-picolylchloride hydrochloride (1.30 g, 7.92 mmol, 2.2 eq.) was then slowly added to the pink suspension. After stirring for 36 h at 80 °C the solvent was removed and the residue was purified via silica gel chromatography (CH_2Cl_2–MeOH 10:1) with the desired product running at the front. **3** was obtained as dark yellow oil (1.24 g, 74%).[6]

^{1}H NMR (300 MHz, $CDCl_3$): δ 8.52 (ddd, J = 1.3 Hz, 1.8 Hz, 5.7 Hz, 2H), 7.66 (m, 4H), 7.49 (d, J = 7.6 Hz, 2H), 7.29 (d, J = 8.1 Hz, 2H) 7.16 (ddd, J = 1.8 Hz, 5.7 Hz, 7.6 Hz, 2H) 3.88 (s, 4H), 3.25 (m, 4H), 3.16 (m, 4H), 2.81 (s, 4H), 2.42 (s, 3H).

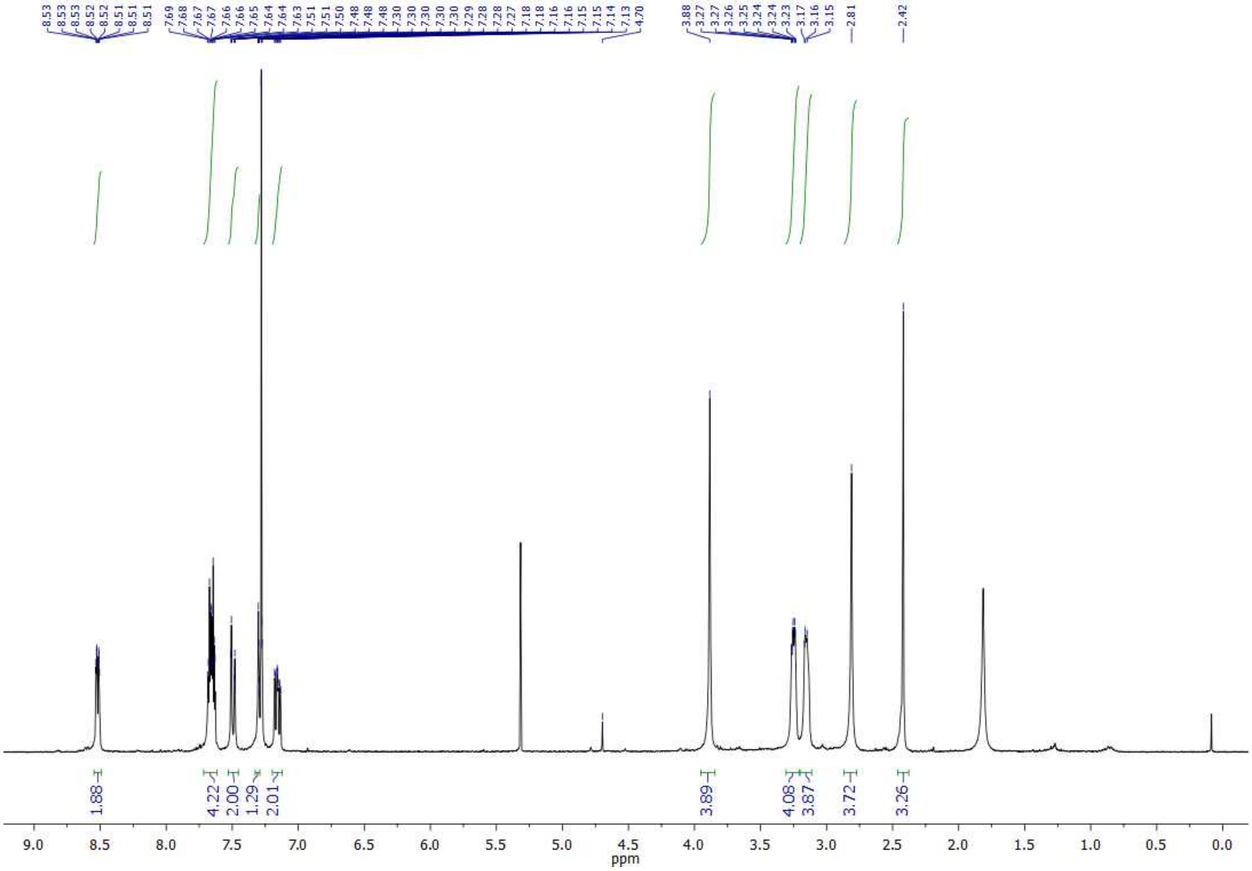

Figure S3: ^{1}H NMR spectrum (300 MHz) of **3** in $CDCl_3$.

2.1.4 Synthesis of 1,4-Di(picolyl)-1,4,7-trazacyclononane (4)

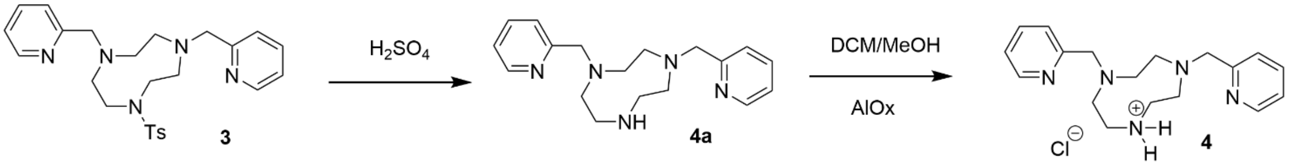

3 (1.42 g, 3.00 mmol) was combined with 10 mL of conc. H_2SO_4 and heated for 24 h at 150 °C. After cooling to 0 °C the black solution was basified to pH ~ 13 using saturated NaOH solution and extracted with vast amount of $CHCl_3$ to yield **4a**. The organic phases were combined, the solvent removed (^{1}H NMR spectrum of crude product in Figure S5) and the residue purified using gel chromatography on aluminum oxide (CH_2Cl_2–MeOH 98:2, third and fourth fraction). The combined fractions were dried over Mg_2SO_4 and the solvent was removed to yield **4** as a dark brown oil (0.76 g, 80%).[7] During the chromatographic work-up process **4a** became protonated as indicated in Figure S4, where a NH signal at 10.5 ppm is observable. The proton source is currently not unambiguously identified; we believe that trace impurities in CH_2Cl_2 are the most likely origin.

^{1}H NMR (300 MHz, $CDCl_3$): δ 10.48 (bs, 1H, NH), 8.64 (d, *J* = 4.3 Hz, 2H), 7.57 (dt, *J* = 1.7, 7.6 Hz, 2H), 7.19 (d, *J* = 7.6, 4H), 3.91 (s, 4H), 3.19 (t, *J* = 5.8 Hz, 4H), 2.99 (t, *J* = 5.8 Hz, 4H), 2.73 (s, 4H).

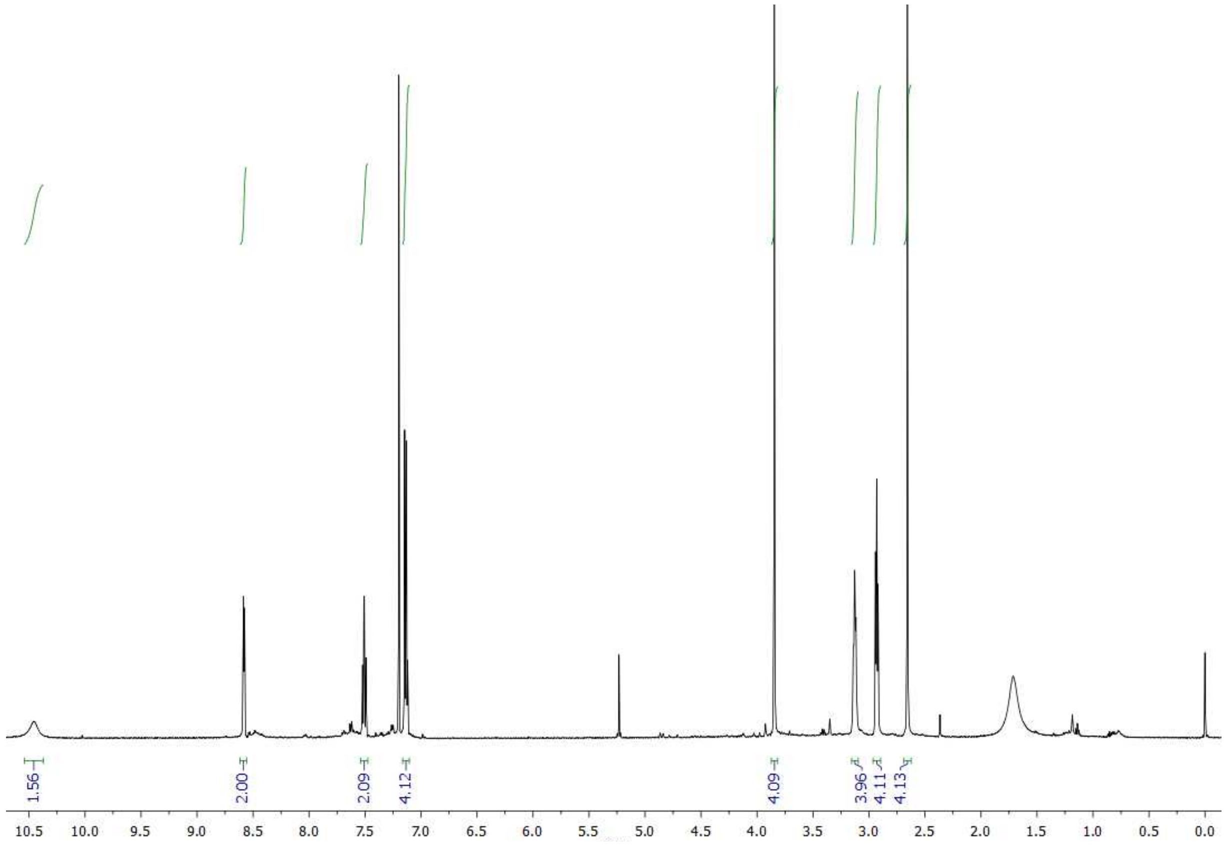

Figure S4: ^{1}H NMR spectrum (300 MHz) of **4** after column chromatography in $CDCl_3$.

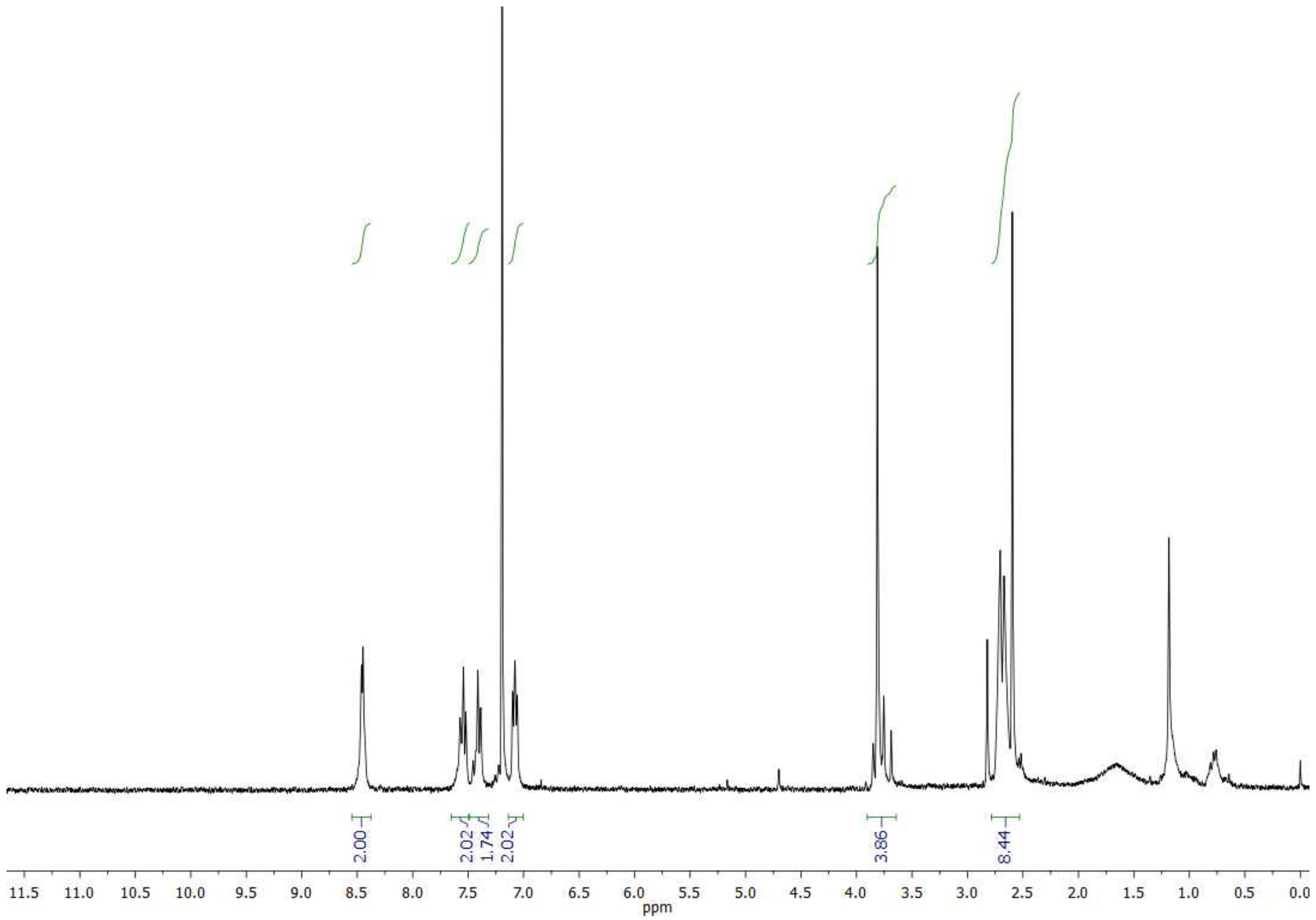

Figure S5: ^{1}H NMR spectrum (300 MHz) of crude **4a** in $CDCl_3$ (δ = 8.45, 7.54, 7.40, 7.08, 3.78, 2.66 ppm).

2.1.5 Synthesis of Bis(2-aminoethyl)sulfide (5)

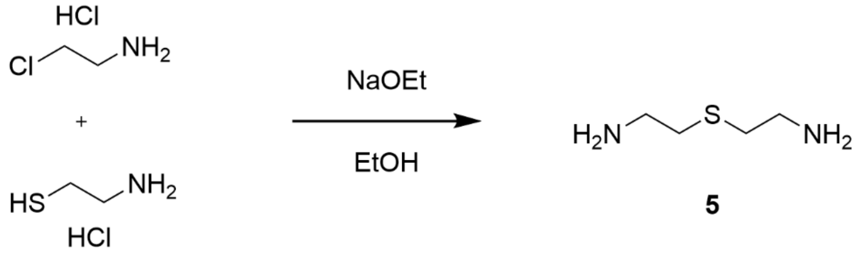

Cysteamine hydrochloride (2.00 g, 17.20 mmol, 1 eq.) was added to a stirred solution of sodium ethanolat (1.20 g, 51.70 mmol, 3 eq.) in ethanol and heated under reflux for 30 min. 2-chloroethylamine hydrochloride (1.96 g, 17.20 mmol, 1 eq.) was added and the reaction mixture was heated for a further 2 h at reflux. The reaction mixture was cooled, the precipitated sodium chloride filtered off and the filtrate concentrated. **5** was yielded as pale yellow oil (1.84 g, 89%).[8]

^{1}H NMR (300 MHz, $CDCl_3$): δ 2.88 (t, J = 6.3 Hz, 4H), 2.62 (t, J = 6.3 Hz, 4H), 1.93 (br, 4H).

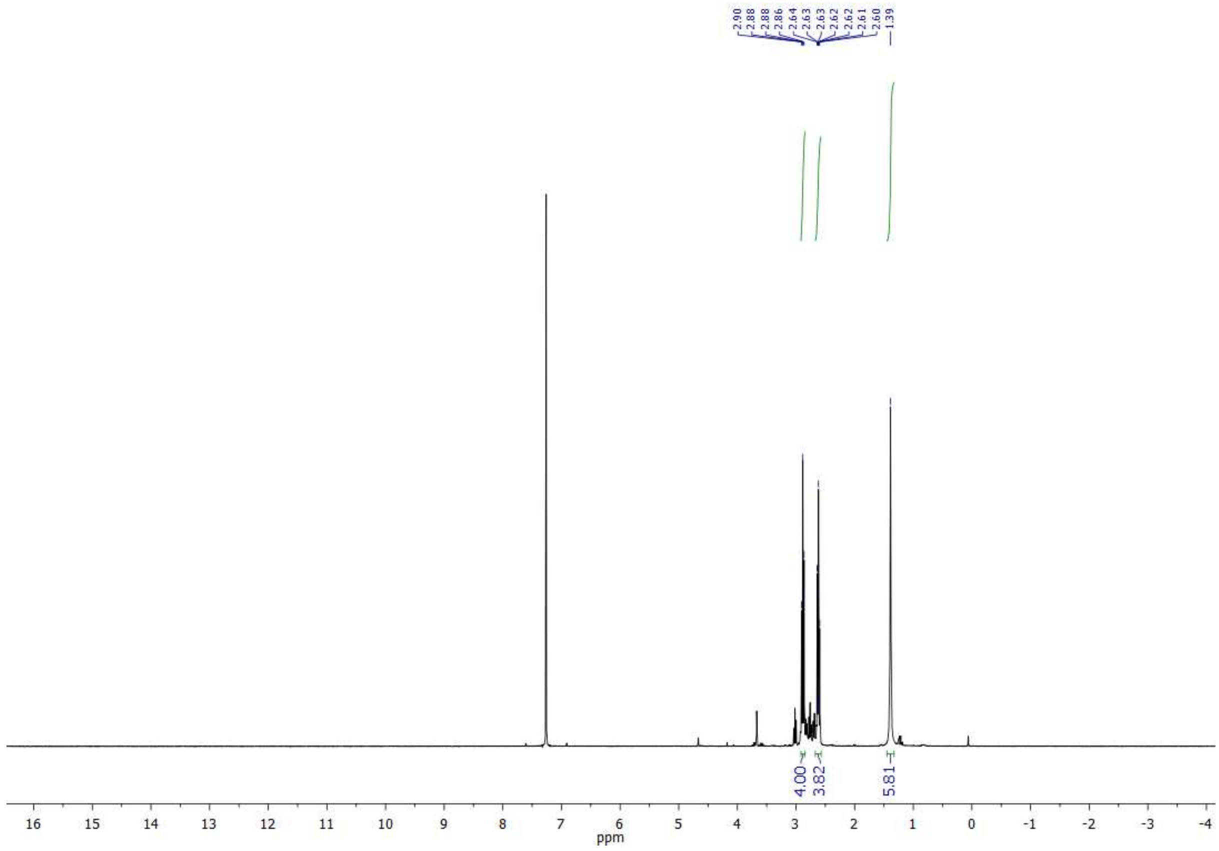

Figure S6: ^{1}H NMR spectrum (300 MHz) of **5** in $CDCl_3$.

2.1.6 Synthesis of N,N'-bis-tosyl-bis(2-aminoethyl)sulfide (6)

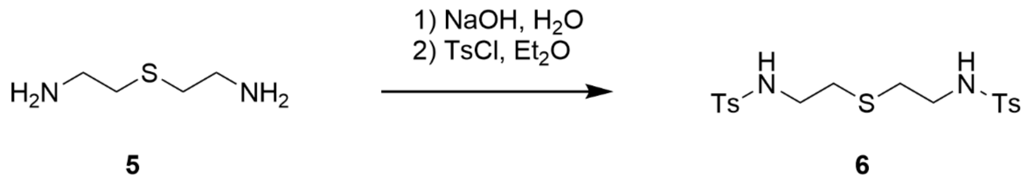

5 (1.84 g, 15.3 mmol, 1 eq.) was combined with NaOH (1.29 g, 32.1 mmol, 2.1 eq.) and dissolved in water (35 mL). TosylCl (6.13 g, 32.1 mmol, 2.1 eq.) was first dissolved in Et_2O (30 mL) and then slowly dropped into the solution. The resulting mixture was vigorously stirred for 36 h at room temperature. Afterwards, the layers were separated and the aqueous layer extracted with $CHCl_3$. The combined organic fractions were reduced to dryness. the residue was purified via silica gel chromatography (CH_2Cl_2–MeOH 9:1) yielding **6** as a bright yellow oil (6.00 g, 91%).[5]

^{1}H NMR (300 MHz, $CDCl_3$): δ 7.67 (d, J = 8.2 Hz, 4H), 7.25 (d, J = 8.2 Hz, 4H), 4.83 (t, J = 6.3 Hz, 2H, NH), 3.01 (q, J = 6.3 Hz, 4H), 2.48 (t, J = 6.3 Hz, 4H), 2.37 (s, 6H).

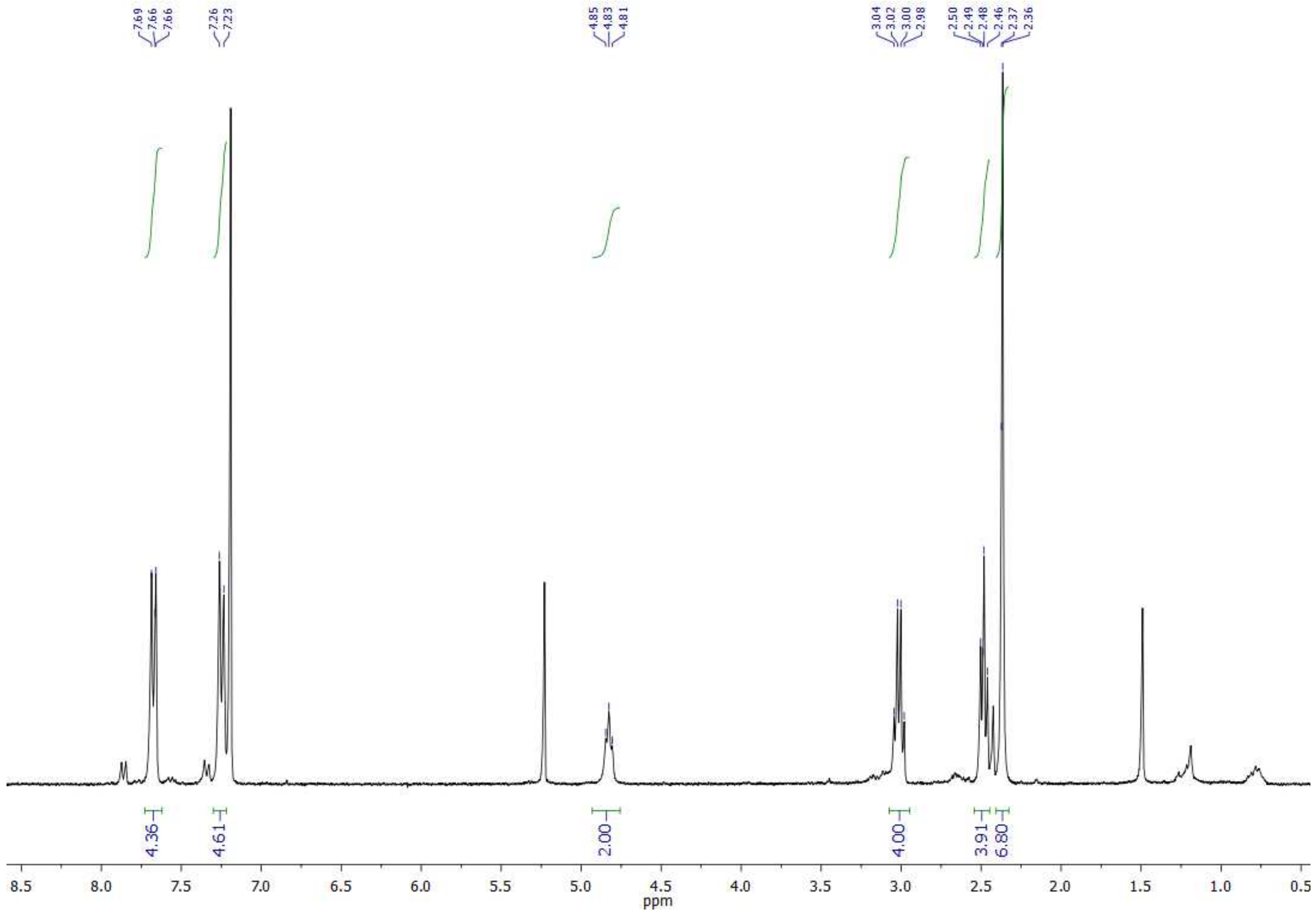

Figure S7: ^{1}H NMR spectrum (300 MHz) of **6** in $CDCl_3$.

2.1.7 Synthesis of 4,7-Bis-tosyl-1-thia-4,7-diazacyclononane (7)

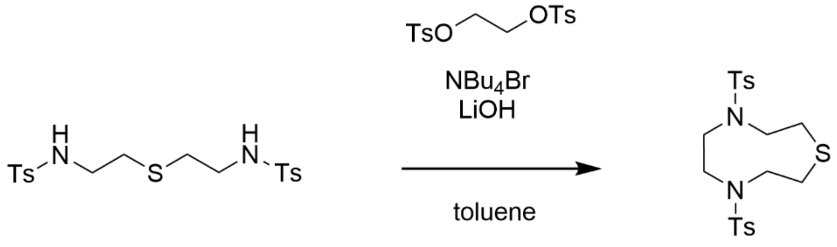

6 **7**

1.88 g (78 mmol) of LiOH were dissolved in 75 ml water and combined with tetrabutylammonium bromide (460.7 mg, 1.42 mmol, 0.25 eq.) in 400 mL toluene and heated under reflux. To this solution di-tosyl-ethylene glycol (2.12 g, 5.72 mmol, 1 eq.) was added in small portions under heavy stirring. To this mixture **6** (2.45 g, 5.72 mmol, 1 eq.), dissolved in toluene (150 mL), was slowly added using a dropping funnel and afterwards stirred under reflux for 12 h. After cooling, the layers were separated and the organic phase was evaporated. The obtained residue was recrystallized in hot MeOH to yield **7** as a colourless solid (1.4 g, 54%).[9]

^{1}H NMR (300 MHz, $CDCl_3$): δ 7.67 (d, *J* = 8.1 Hz, 4H), 7.33 (d, *J* = 8.1 Hz, 4H), 3.49 (t, *J* = 4.7 Hz, 4H), 3.37 (s, 4H), 3.13 (t, *J* = 4.7 Hz, 4H), 2.44 (s, 6H).

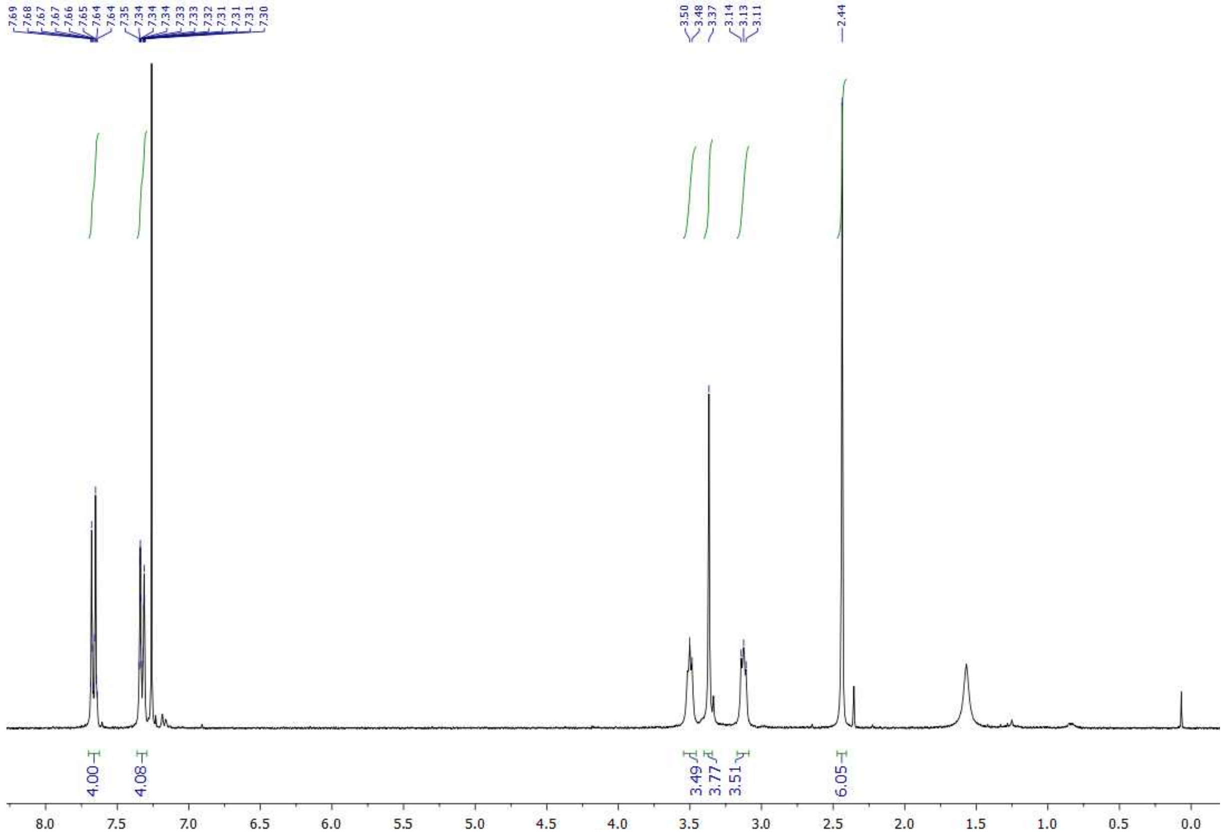

Figure S8: ^{1}H NMR spectrum (300 MHz) of **7** in $CDCl_3$.

2.1.8 Synthesis of 1-Thia-4,7-diazacyclononane (8)

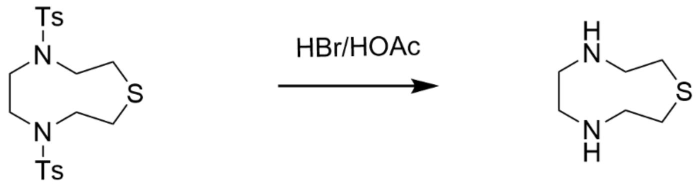

7 **8**

7 (2.70 g, 5.94 mmol) was dissolved in 50 mL of HBr in glacial acetic acid (33%) and stirred for 3 days. After completion, the mixture was concentrated by removing acetic acid as azeotropic mixture using toluene (4x20 mL). The crude product was dissolved in water and washed with CH_2Cl_2. The aqueous layer was concentrated, redissolved in distilled water and 10 eq. of 1 M NaOH were added. The aqueous phase was removed using a Dean-Stark-apparatus by using toluene, from which **8** was obtained as a pale yellow oil (160 mg, 18%) after reduction to dryness.[9]

^{1}H NMR (300 MHz, $CDCl_3$): δ 2.88 (m, 4H), 2.72 (m, 8H), 2.08 (br, 2H).

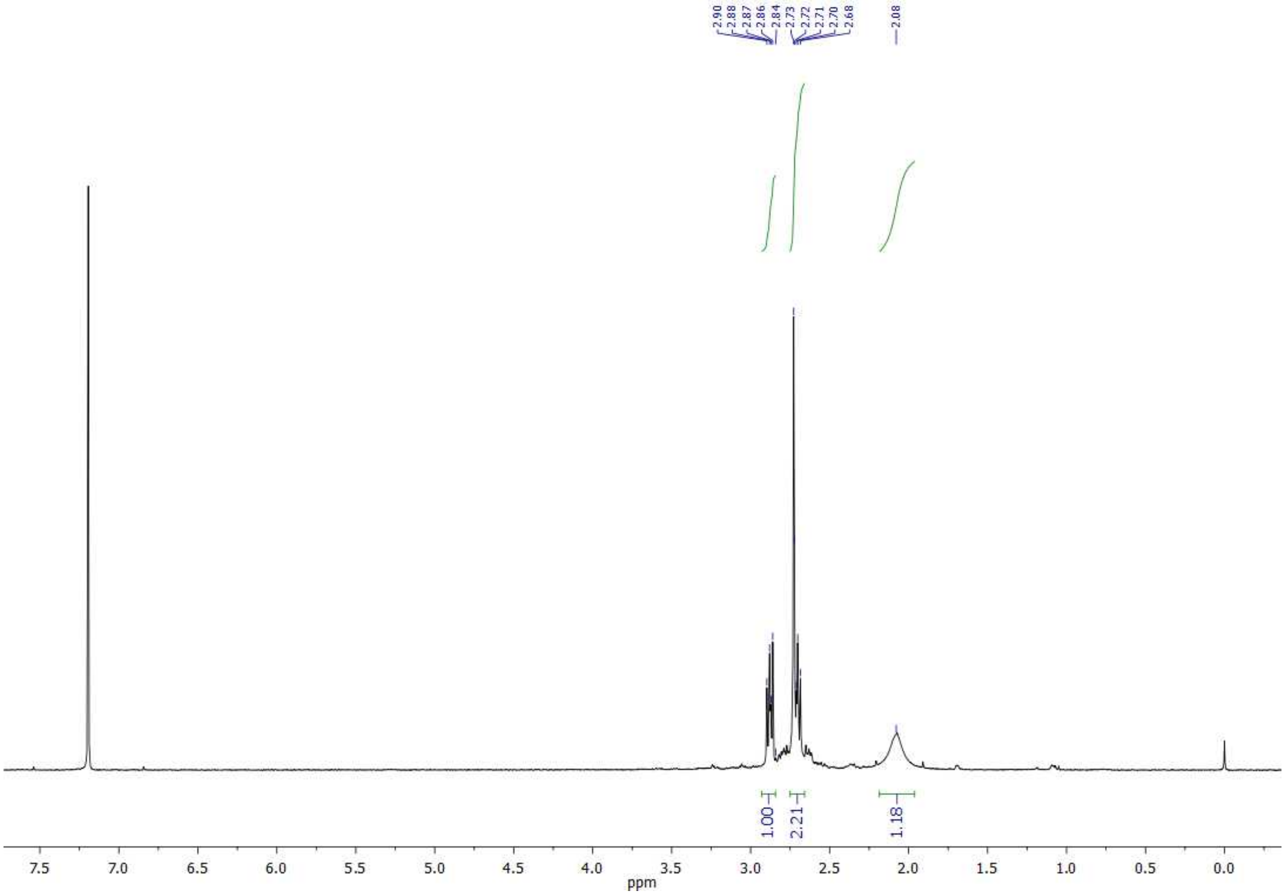

Figure S9: ^{1}H NMR spectrum (300 MHz) of **8** in $CDCl_3$.

2.1.9 Synthesis of 4,7-Di(picolyl)-1-thia-4,7-diazacyclononane (9)

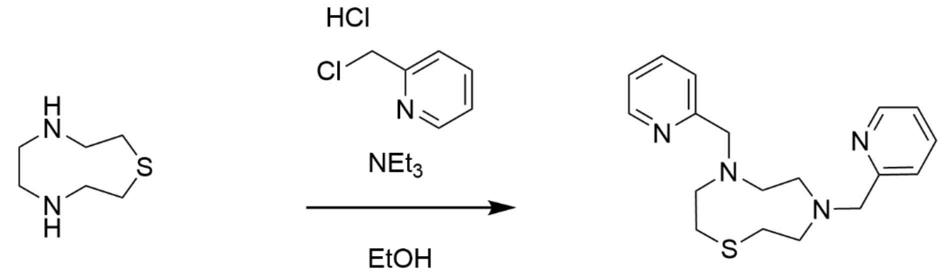

8 **9**

8 (160 mg, 1.09 mmol, 1 eq.) and 2-picolylchloride hydrochloride (359 mg, 2.19 mmol, 2 eq.) were dissolved in absolute EtOH (20 mL) and NEt_3 (1.22 ml, 8.75 mmol, 8 eq.) was slowly added. The red-turned clear solution was heated under reflux for 16 h and after cooling down washed with water (150 mL). The aqueous phase was extracted three times with $CHCl_3$. Afterwards, the organic phases were combined, dried over $MgSO_4$ and concentrated to yield pure **9** as brown oil (260 mg, 72%).[9]

^{1}H NMR (300 MHz, $CDCl_3$): δ 8.54 (d, J = 4.8 Hz, 2H) 7.59 (t, J = 7.7 Hz, 2H), 7.46 (d, J = 7.7 Hz 2H), 7.09 (t, J = 6.0 Hz, 2H), 3.79 (s, 4H), 3.05 (m, 4H), 2.89 (m, 4H), 2.60 (m, 4H).

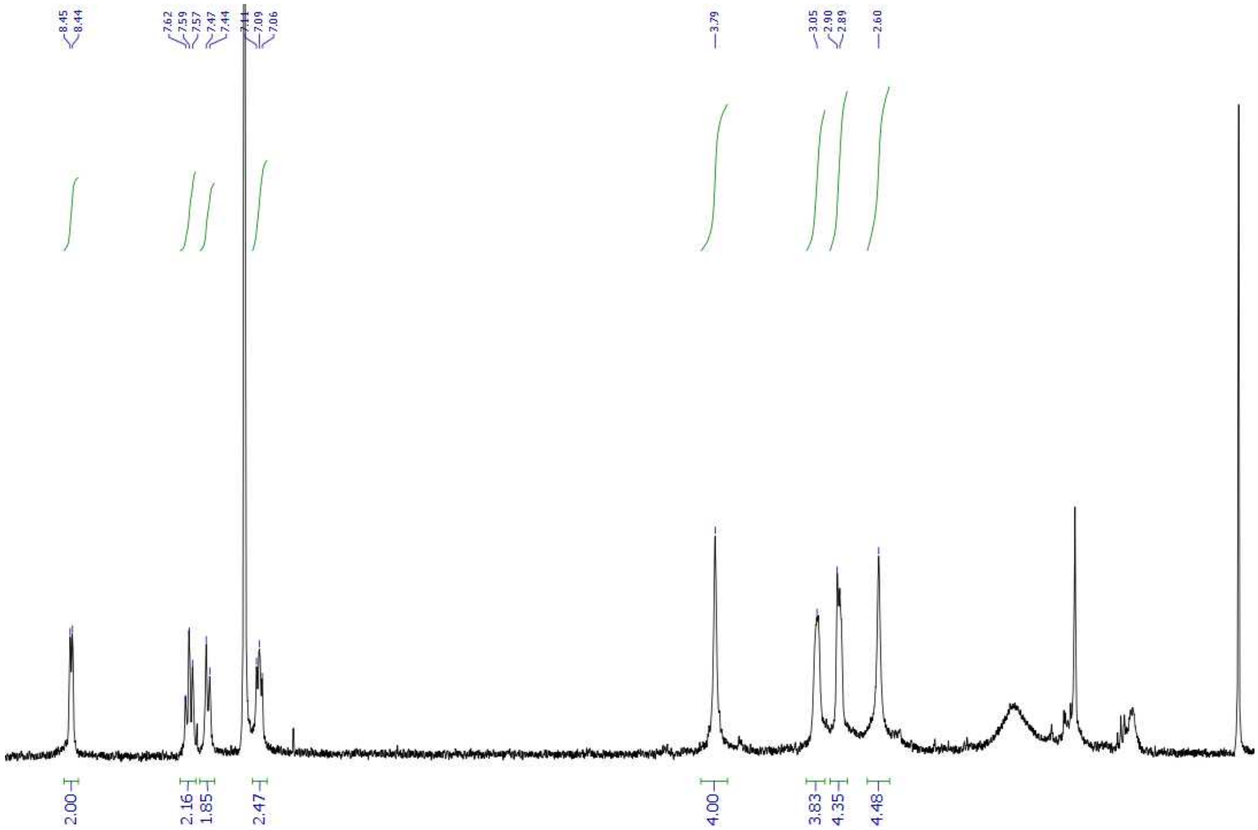

Figure S10: ^{1}H NMR spectrum (300 MHz) of **9** in $CDCl_3$.

2.2 Additional NMR spectra

2.2.1 ^{1}H NMR spectrum of Co-L^{N3}

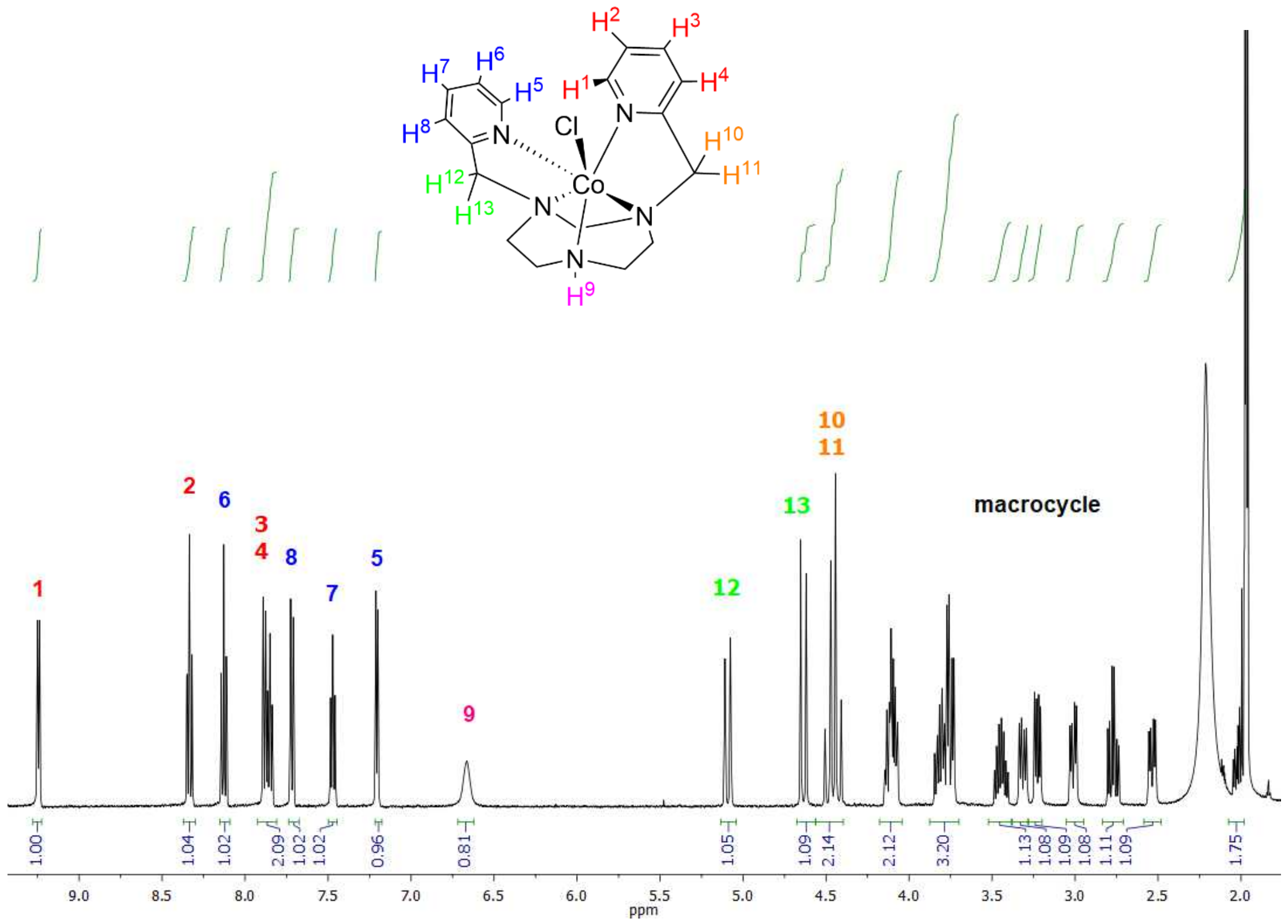

Figure S11: ^{1}H NMR spectrum of **Co-L^{N3}** in MeCN-d^3.

2.2.2 ^{1}H NMR spectrum of Co-L^{N2S}

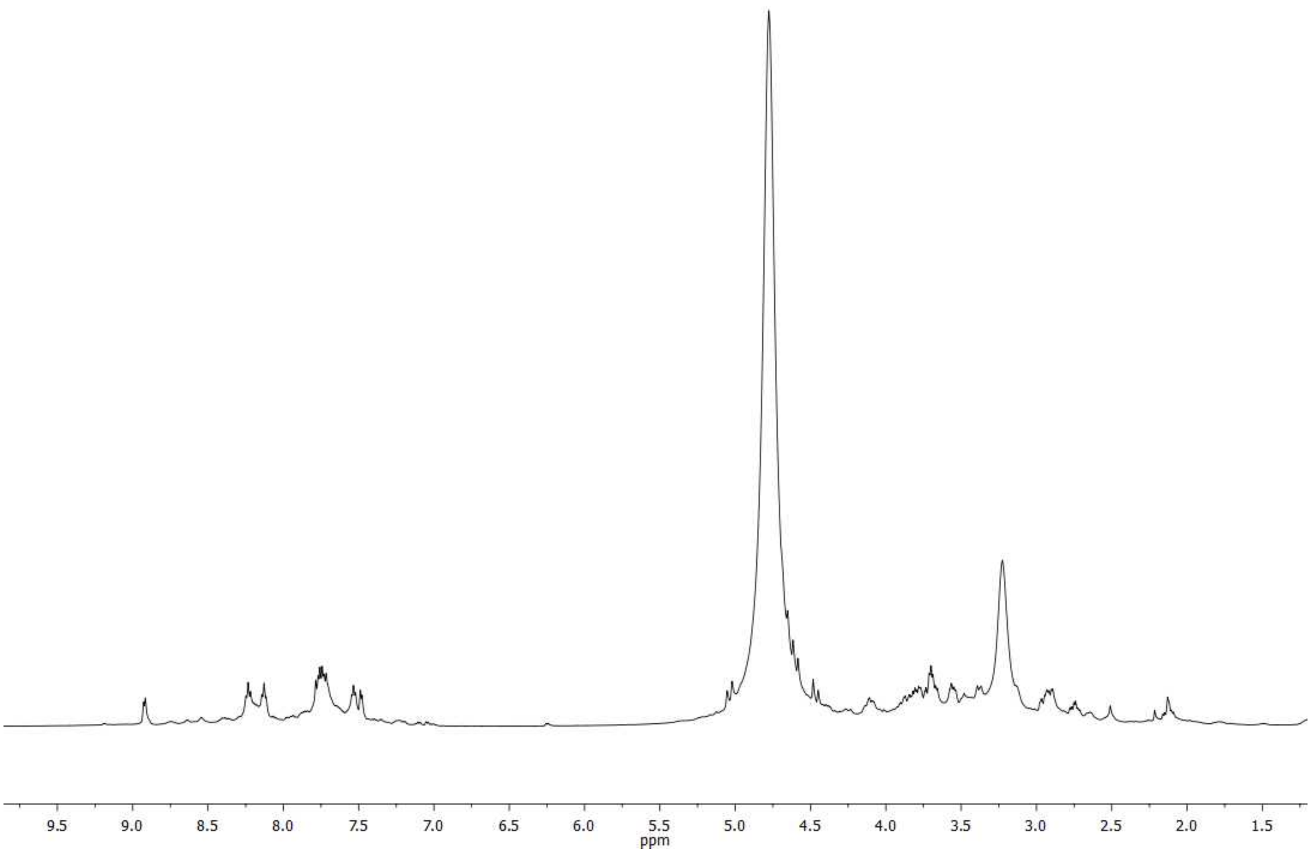

Figure S12: ^{1}H-NMR spectrum of **Co-L^{N2S}** in MeOD.

3 MS spectra

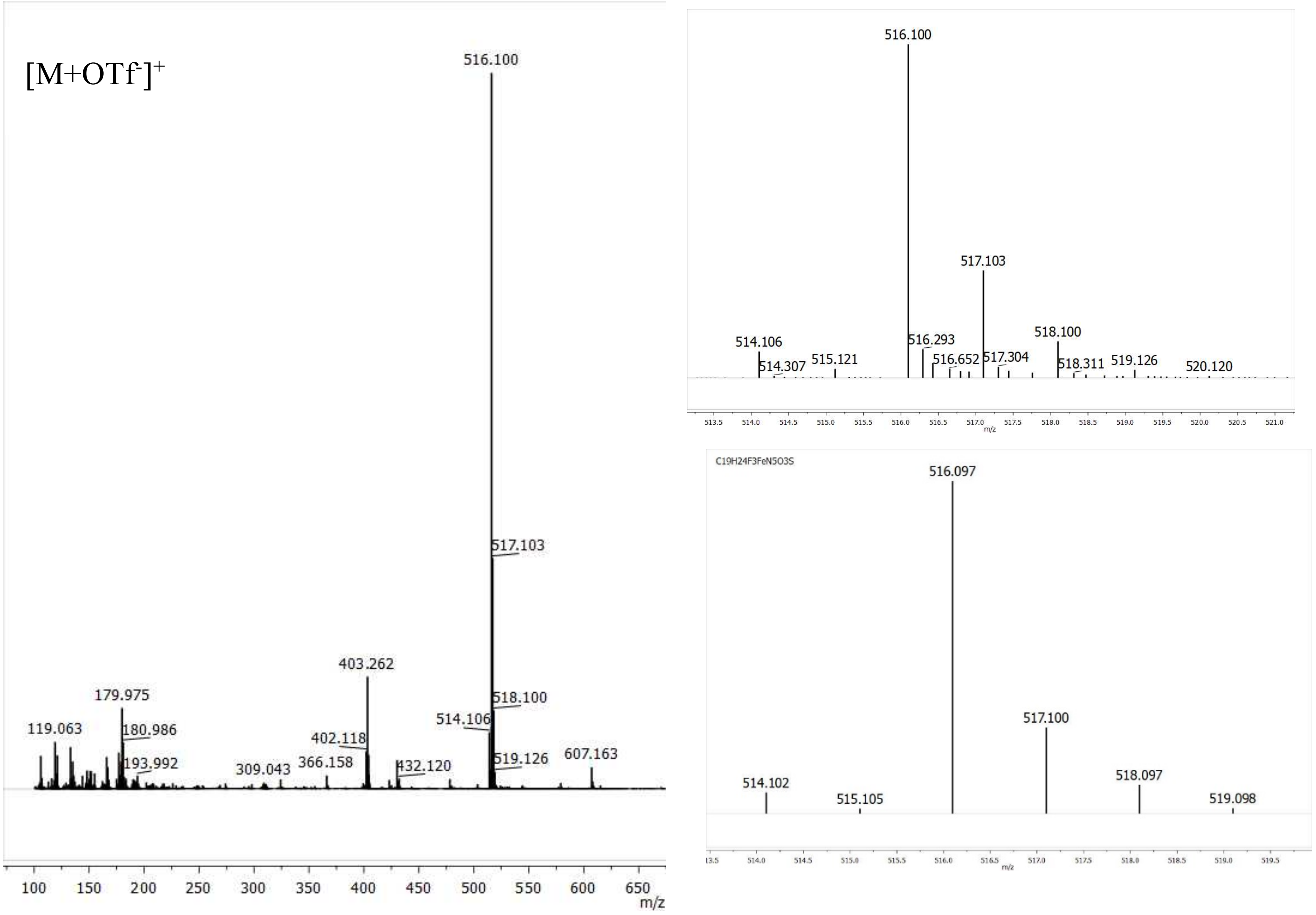

Figure S13: HR-ESI mass spectrum of **Fe-L^{N3}** [$C_{19}H_{25}FeF_3N_5O_3S^+$] and comparison of the measured (top right) to the calculated (bottom right) isotopic pattern of the main peak.

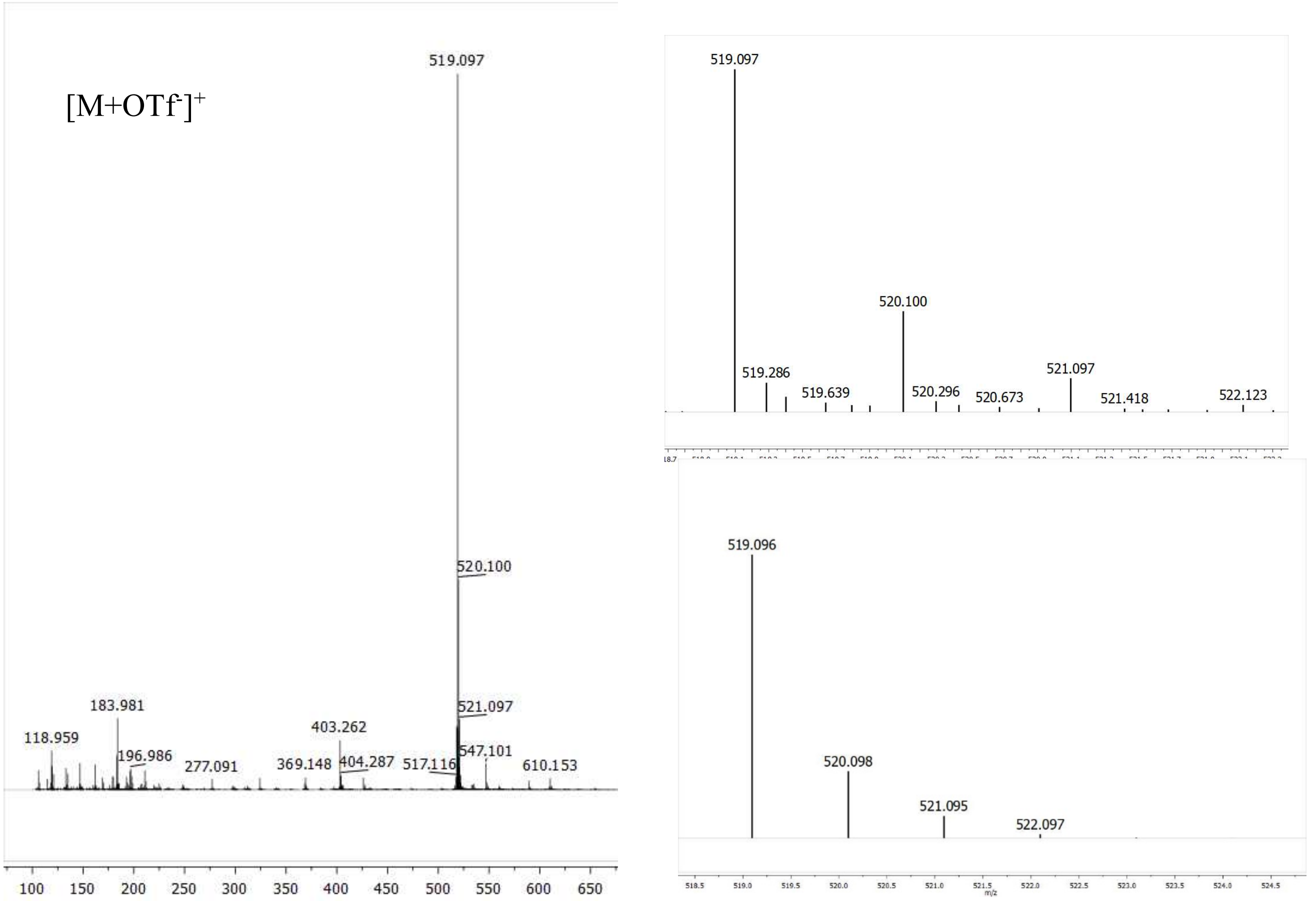

Figure S14: HR-ESI mass spectrum of **Co-L^{N3}** [$C_{19}H_{25}CoF_3N_5O_3S^+$] and comparison of the measured (top right) to the calculated (bottom right) isotopic pattern of the main peak.

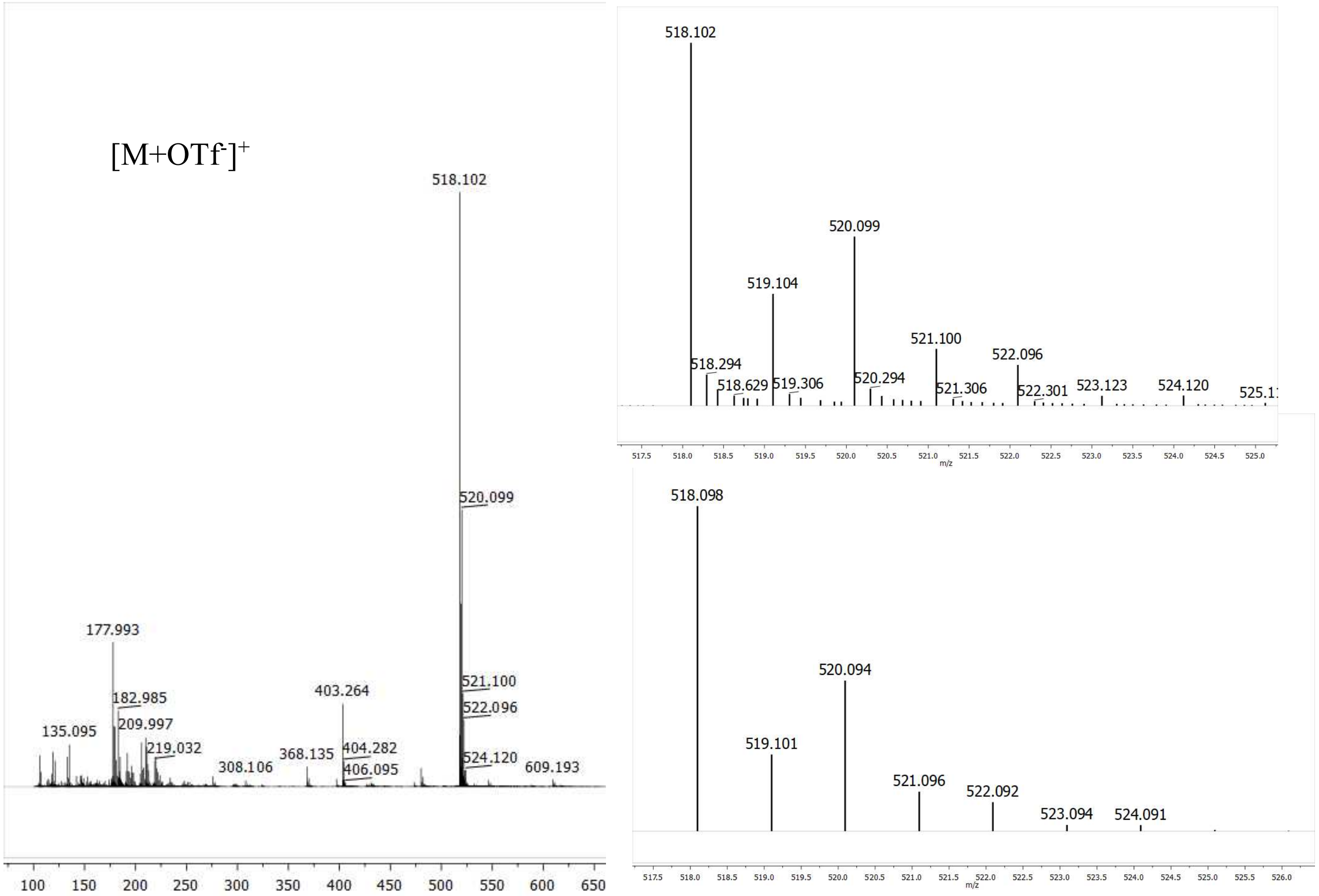

Figure S15: HR-ESI mass spectrum of **Ni-L^{N3}** [$C_{19}H_{25}NiF_3N_5O_3S^+$] and comparison of the measured (top right) to the calculated (bottom right) isotopic pattern of the main peak.

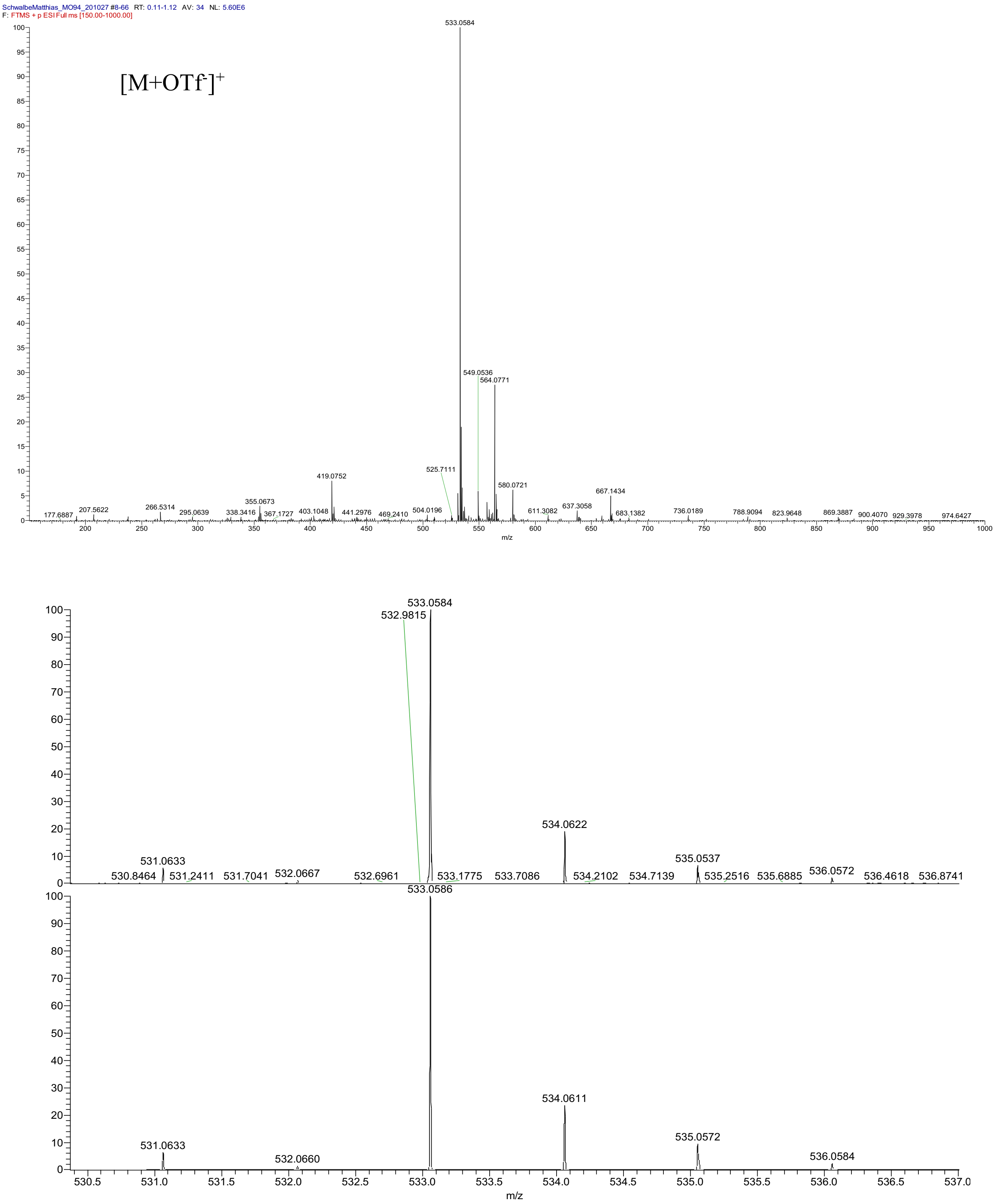

Figure S16: HR-ESI mass spectrum of **Fe-L$^{\mathbf{N2S}}$** [$C_{19}H_{24}FeF_3N_4O_3S_2^+$] (top) and comparison of the measured (middle) to the calculated (bottom) isotopic pattern of the main peak.

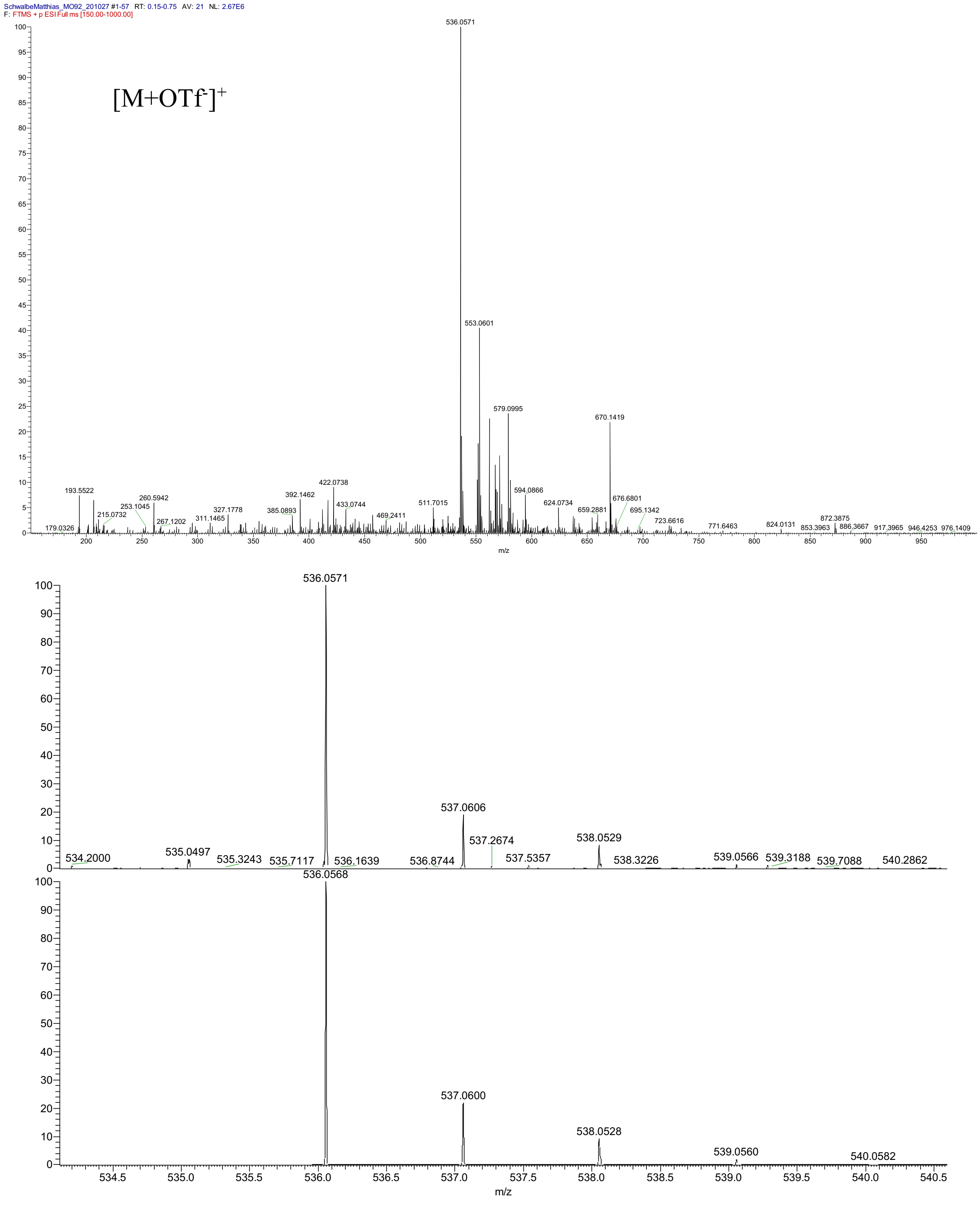

Figure S17: HR-ESI mass spectrum of **Co-L^{N2S}** [$C_{19}H_{24}CoF_3N_4O_3S_2^+$] (top) and comparison of the measured (middle) to the calculated (bottom) isotopic pattern of the main peak.

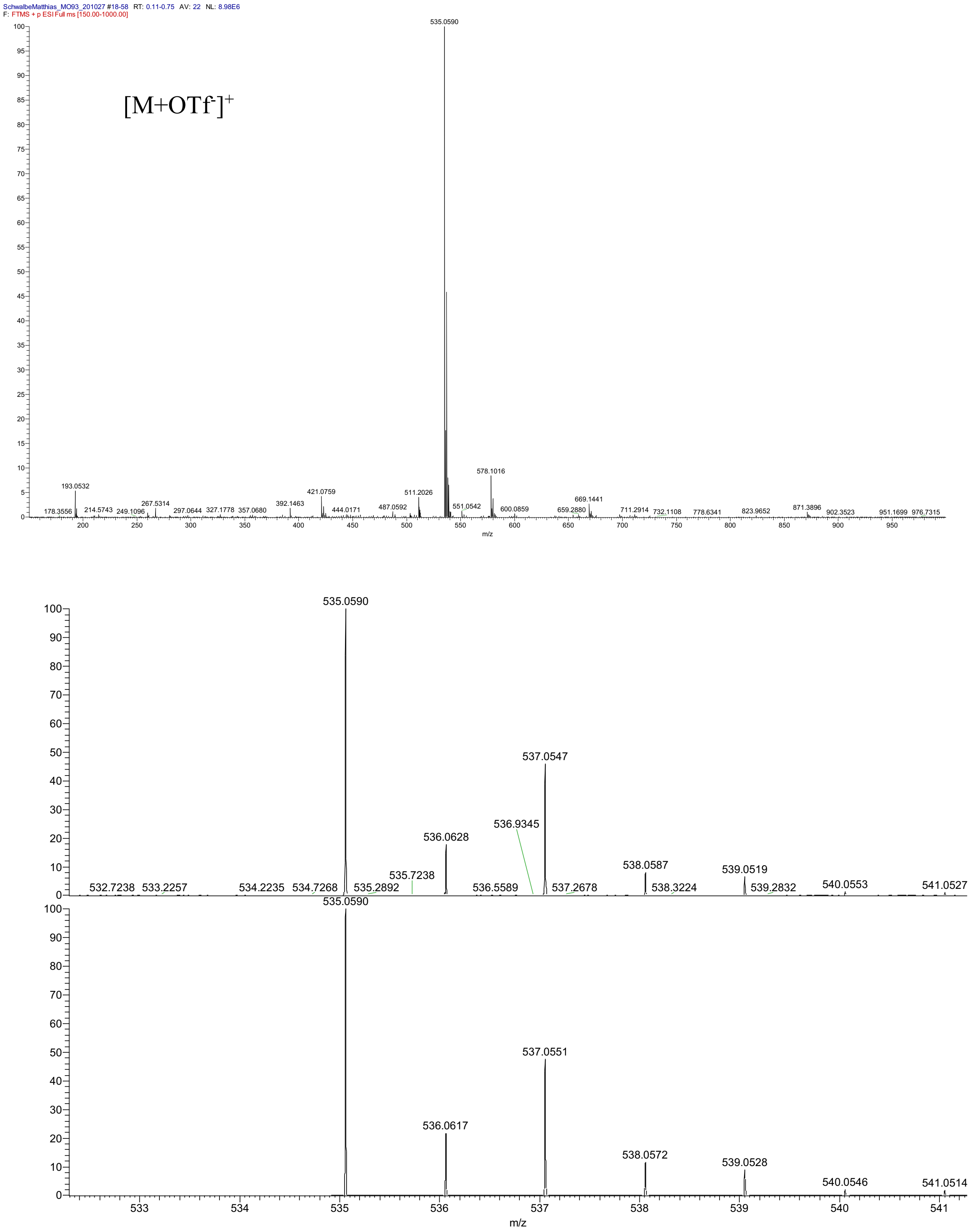

Figure S18: HR-ESI mass spectrum of **Ni-L^{N2S}** [$C_{19}H_{24}NiF_3N_4O_3S_2^+$] (top) and comparison of the measured (middle) to the calculated (bottom) isotopic pattern of the main peak.

4 UV/vis measurements

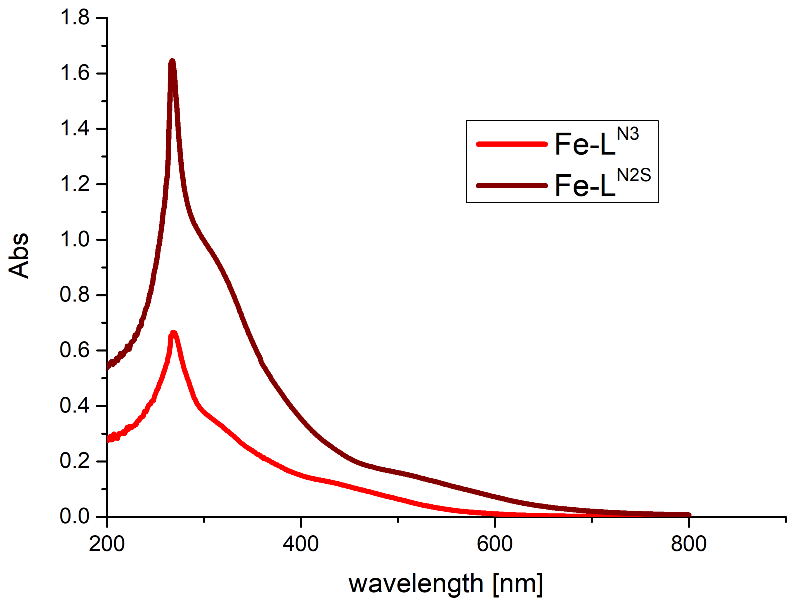

Figure S19: UV/vis spectrum of **Fe-L^{N3}** and **Fe-L^{N2S}** in DMF.

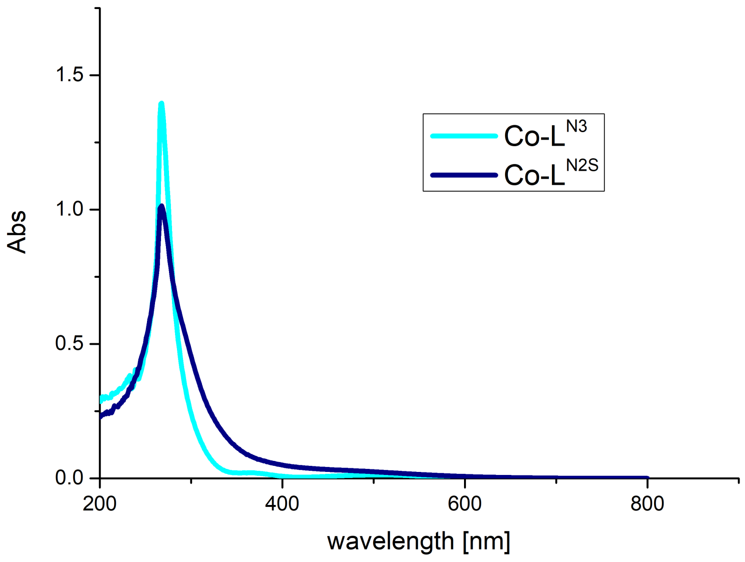

Figure S20: UV/vis spectrum of **Co-L^{N3}** and **Co-L^{N2S}** in DMF.

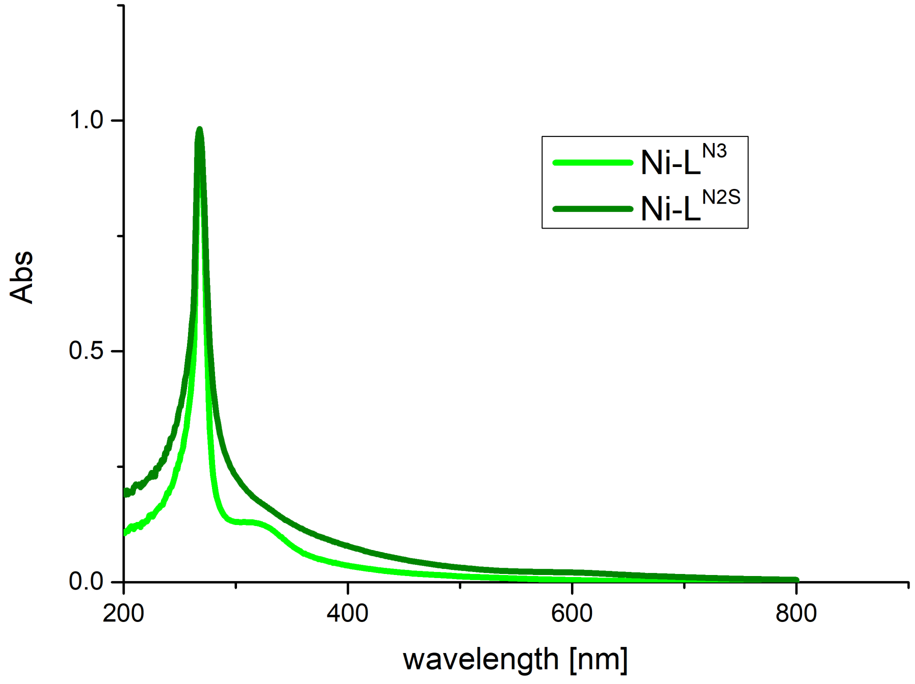

Figure S21: UV/vis spectrum of **Ni-L^{N3}** and **Ni-L^{N2S}** in DMF.

5 CV measurements

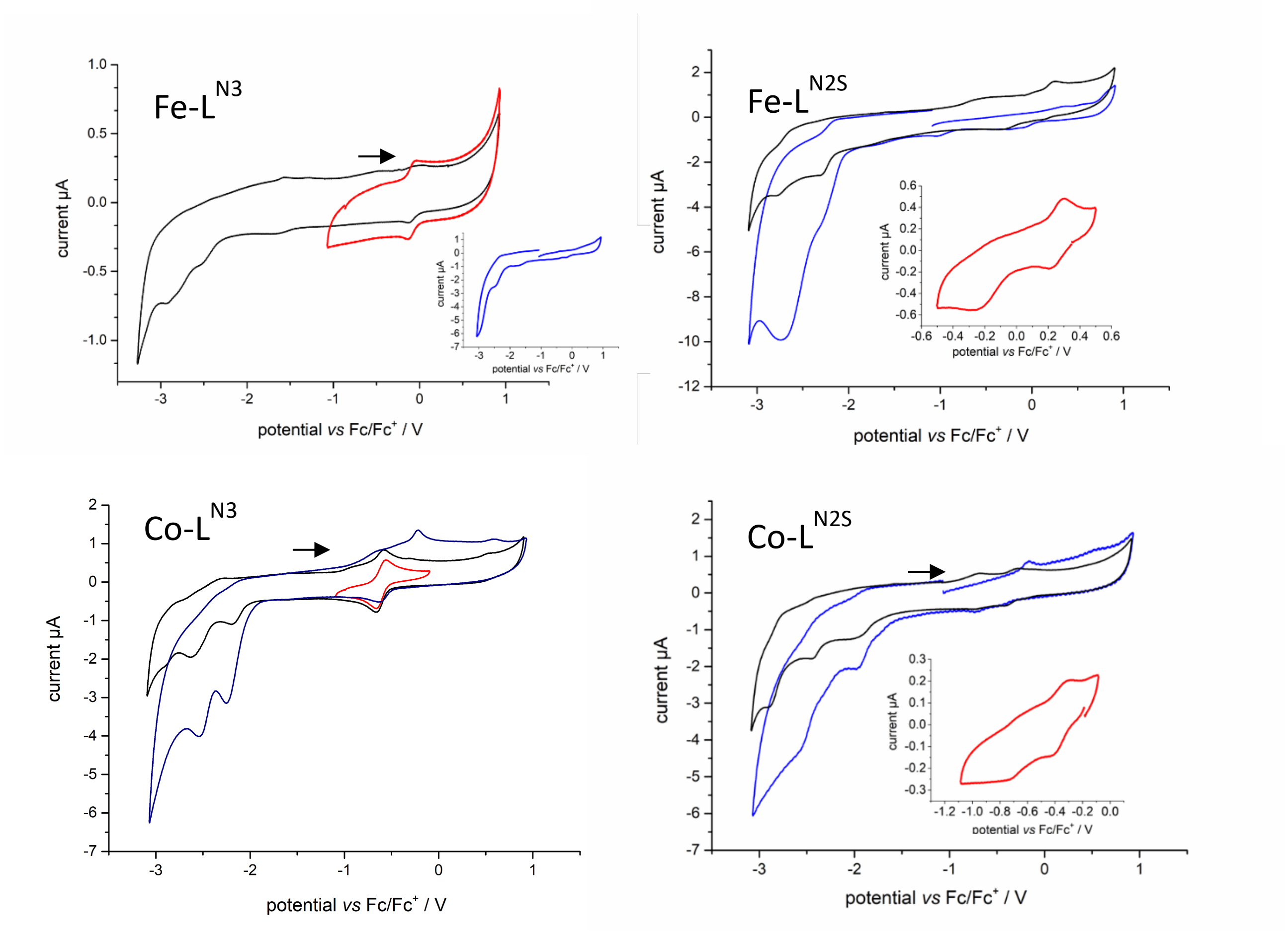

Figure S22: Cyclic voltammograms in DMF under Ar (black) or CO_2 (blue) atmosphere with 0.1 M Bu_4NPF_6 as electrolyte were measured with a scan rate of 0.2 V/s: **Fe-L^{N3}** top left; **Fe-L^{N2S}** top right; **Co-L^{N3}** bottom left; **Co-L^{N2S}** bottom right. In red a narrow scan range focusing on the M^{III}/M^{II} redox couple is shown. (The insets in case of **Fe-L^{N2S}** and **Co-L^{N2S}** show the CV measured with a scan rate of 0.1 V/s; the inset for **Fe-L^{N3}** shows the CV under CO_2 atmosphere: high currents made it necessary to show the CV as inset for better visibility of the redox events under argon atmosphere.)

6 EPR measurements

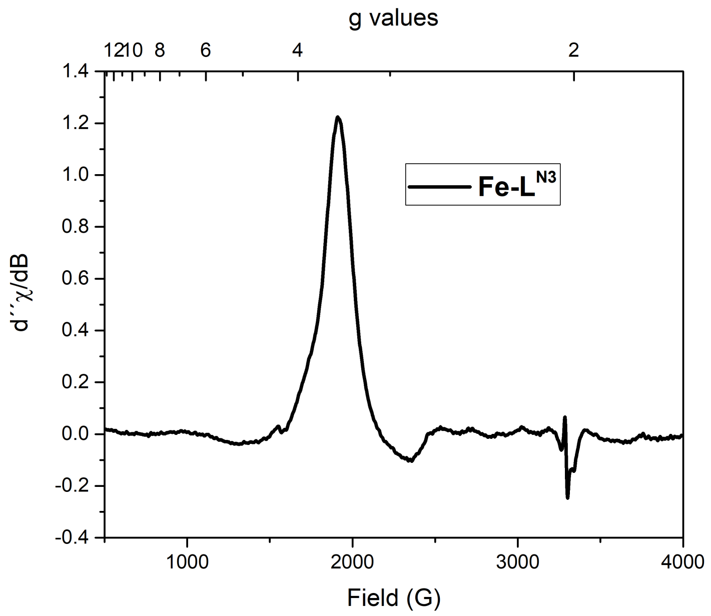

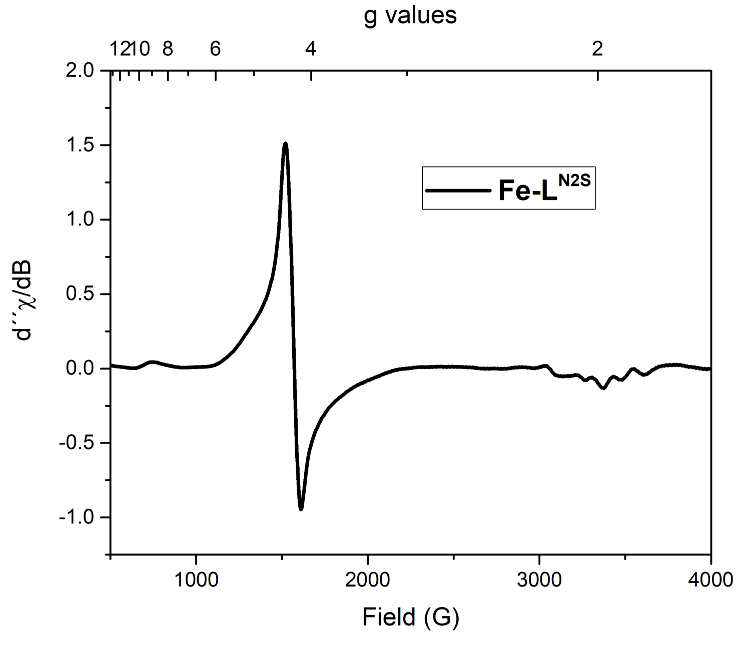

Figure S23: EPR measurements of **Fe-L^{N3}** and **Fe-L^{N2S}** with field in G and the relating *g* values.

7 Crystallographic data

Table S1: Crystallographic data of the compounds **Fe-L^{N3}** and **Co-L^{N3}**.

**compound**	**Fe-L^{N3}**	**Co-L^{N3}**
**CCDC number**	2096076	2096077
**empirical formula**	$C_{20}H_{25}ClFeN_5F_6O_6S_2$	$C_{20}H_{25}ClCoN_5F_6O_6S_2$
**formula weight**	700.87	703.95
**temperature**	100(2) K	100(2) K
**wavelength**	0.71073 / Mo Ka	0.71073 / Mo Ka
**crystal system**	monoclinic	monoclinic
**space group**	$P2_1$/c	$P2_1$/c
**unit cells dimension**	a = 11.5859(3) Å<br>b = 15.4174(4) Å<br>c = 15.2377(4) Å<br>α = γ = 90°<br>β = 97.1990(10)°	a = 11.5663(8) Å<br>b = 15.4036(11) Å<br>c = 15.2212(9) Å<br>α = γ = 90°<br>β = 97.515(2)°
**volume**	2700.37(12) Å^3	2688.6(3) Å^3
**$\rho_{calcd.}$**	1.724 g/cm^3	1.739 g/cm^3
**Z**	4	4
**F(000)**	1428	1428
**theta range for data collection**	2.47 - 25.38°	2.21 – 28.24°
**reflections total**	24993	147345
**independent reflexes**	4958	6701
**reflexes [I>2sigma(I)]**	4375	5463
**goodness-of-fit F^2**	1.043	1.049
**final R indices [I>2sigma(I)]**	R_1 = 0.0267<br>wR_2 = 0.0627	R_1 = 0.0357<br>wR_2 = 0.0748
**R indices (all data)**	R_1 = 0.0331<br>wR_2 = 0.0660	R_1 = 0.0508<br>wR_2 = 0.0803

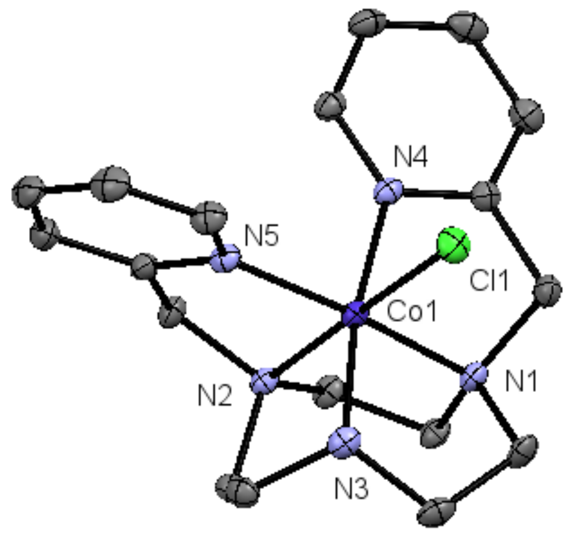

Figure S24: Crystal Structure of **Co-L^{N3}**. Hydrogen atoms and counter anions are omitted for clarity.

Table S2: Selected interatomic distances (Å) and bond angles (°) for **Co-L^{N3}**.

Co(1)-Cl(1)	2.239(6)	N(2)-Co(1)-Cl(1)	175.52(5)
Co(1)-N(1)	1.945(17)	N(3)-Co(1)-N(4)	171.11(7)
Co(1)-N(2)	1.960(17)	N(5)-Co(1)-N(1)	171.81(7)
Co(1)-N(3)	1.937(17)	N(2)-Co(1)-N(5)	84.19(7)
Co(1)-N(4)	1.967(17)	N(3)-Co(1)-Cl(1)	89.66(5)
Co(1)-N(5)	1.944(17)	N(4)-Co(1)-N(1)	84.14(7)

8 Photocatalytical and photophysical studies

Table S4: TON for **Co-L^{N2S}** after 24 h irradiation with 1eq. **Ir** for different ratios (Vol-%) of TEA and H_2O in DMF.

ratio TEA:H_2O	*TON_{CO}*	*TON_{H2}*
5:0	30	16
5:1	18	24
5:5	5	54

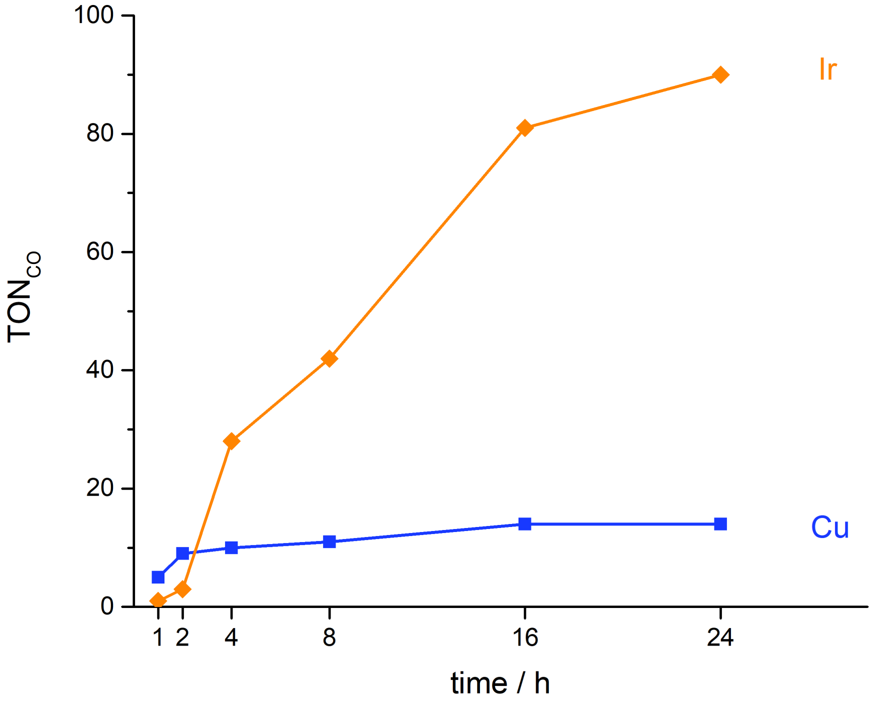

Figure S25: TON_{CO} for **Co-L^{N3}** in dependence of the used PS (blue: **Cu**; orange: **Ir)** for different times of irradiation.

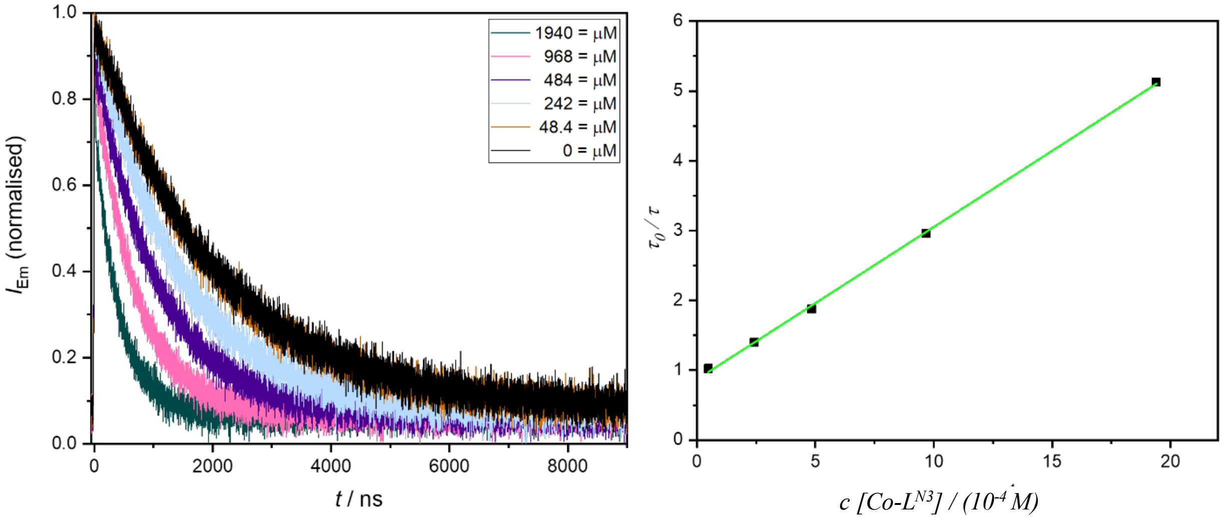

Figure S26: (left) Luminescence quenching studies in acetonitrile of **Ir** with increasing amount of **Co-L^{N3}** ($\lambda_{ex.}$ = 355 nm, $\lambda_{detec.}$ = 490 nm); (right) corresponding Stern-Volmer analysis leading to K_{SV} = 2185 M^{-1} and k_q = 1.084 x 10^9 $M^{-1}s^{-1}$.

References

[1] G. M. Sheldrick, *SADBAS* **1996**, University of Göttingen, Germany.

[2] G. M. Sheldrick, *Acta Crystallogr. A Found Adv.* **2015**, *71*, 3-8.

[3] G. M. Sheldrick, *Acta Crystallogr. C Struct. Chem.* **2015**, *71*, 3-8.

[4] F. Friscourt, C. J. Fahrni, G. J. Boons, *J. Am. Chem. Soc.* **2012**, *134*, 18809-18815.

[5] R. Cao, P. Müller, S. J. Lippard, *J. Am. Chem. Soc.* **2010**, *132*, 17366-17369.

[6] V. Stavila, M. Allali, L. Canaple, Y. Stortz, C. Franc, P. Maurin, O. Beuf, O. Dufay, J. Samarut, M. Janier, J. Hasserodt, *New J. Chem.* **2008**, *32*, 428-435.

[7] N. G. Luk'yanenko, S. S. Basok, L. K. Filonova, N. V. Kulikov, V. N. Pastushok, *Chem. Heterocycl. Comp.* **1990**, *26*, 346-349.

[8] J. Wilson, **2007**, University of Glasgow, Great Britain.

[9] B. Chak, A. McAuley, T. W. Whitcombe, *Can. J. Chem.* **1994**, *72*, 1525-1532.
